# Phylogeny and historical biogeography of leafhopper subfamily Evacanthinae (Hemiptera: Cicadellidae) based on morphological and molecular data

**DOI:** 10.1038/srep45387

**Published:** 2017-04-03

**Authors:** Yang Wang, Christopher H. Dietrich, Yalin Zhang

**Affiliations:** 1 Key Laboratory of Plant Protection Resources and Pest Management, Ministry of Education, Entomological Museum, Northwest A&F University, Yangling, Shaanxi 712100, China; 2 Illinois Natural History Survey, Prairie Research Institute, University of Illinois, 1816 S Oak St., Champaign, Illinois 61820, USA

## Abstract

Phylogenetic relationships among major lineages of the Evacanthinae, a highly diverse leafhopper subfamily distributed worldwide, were explored by analysing a dataset of 100 discrete morphological characters and DNA sequence data from five gene regions. Sixty-seven taxa representing all evacanthine tribes and all regional faunas, and fourteen putative outgroup taxa were included. Maximum-likelihood and Bayesian analyses yielded similar tree topologies that were well resolved with strong support for the monophyly of Evacanthinae and its four previously included tribes, but indicated that *Draconirvana* Dietrich, was incorrectly placed to tribe and that *Sophonia* Walker, *Evacanthus* Le Peletier & Serville, *Bundera* Distant, *Paraonukia* Ishihara and *Onukia* Matsumura are not monophyletic. Divergence time analysis suggests that the deepest divergences coincided with breakup of Gondwana but that more recent divergences occurred largely within a single biogeographic realm during the Paleogene, with a few long-distance dispersal events. Biogeographical analyses suggest that Evacanthinae originated in Neotropical region. A new tribe, Pentoffiini **trib.n.,** is established to include *Pentoffia* Kramer and *Evanirvana* Hill, the genus *Draconirvana* Dietrich, **placement n.** is transferred to Evacanthini from Nirvanini, a key to tribes is also given and illustrations of representative genera are provided.

Leafhoppers (Cicadellidae), comprising more than 22,000 described species, are one of the largest insect families and inhabit nearly all habitats that support vascular plants worldwide, from tropical rainforest to arctic tundra. Knowledge of leafhopper phylogeny remains rudimentary overall, although some recent studies based on morphology and/or molecular data have yielded estimates of relationships among leafhoppers as a whole^1^,^2^,^3^ as well as within certain cicadellid subfamilies^4^,^5^,^6^,^7^. These recent studies have resulted in some changes to the higher classification of the family with a reduction of recognized subfamilies from 40^8^ to 25 at present^3^,^6^. Improved knowledge of leafhopper phylogeny is needed not only to test the phylogenetic status of currently recognized higher taxa but also to elucidate the evolutionary factors that promoted diversification in this highly successful and economically important group of herbivores.

One diverse leafhopper group that remains poorly studied is the subfamily Evacanthinae^9^, which comprises 513 described species in 71 genera so far and is distributed worldwide^10^, primarily inhabiting humid forest habitats. The classification of this group has been unstable over the past several decades with tribes presently included in Evacanthinae previously placed in the separate subfamilies Cicadellinae (tribe Evacanthini Metcalf) and Nirvaninae (tribes Nirvanini Baker and Balbillini Baker)^8^ and united with unrelated groups (e.g., Occinirvanini Evans, presently included in Deltocephalinae). A previous molecular phylogenetic analysis of Cicadellidae recovered the included representatives of Evacanthinae (in the present sense) as a monophyletic group^2^. Based on a subsequent phylogenetic analysis of adult morphological characters, Dietrich confirmed the monophyly of Evacanthinae comprising tribes Nirvanini, Balbillini, Evacanthini and Pagaroniini Anufriev^9^.

Tribe Evacanthini includes one genus that occurs throughout the Holarctic region but is otherwise restricted to the Oriental region, particularly in southern China and the Indochinese Peninsula. There have been many genera and species of Evacanthini described from this area recently^11^,^12^,^13^,^14^,^15^,^16^,^17^,^18^. Although 27 evacanthine genera have been reported so far, no phylogenetic study of these genera has been made and a comprehensive taxonomic revision is needed. Pagaroniini are restricted to the temperate zone of the Pacific rim from Japan, Korea, and the Russian Far East to the western USA^19^.

Tribe Nirvanini is the most diverse and widespread group of Evacanthinae. Species of this tribe are dorsoventrally flattened leafhoppers distributed throughout the tropical regions of the world. The group includes a few agricultural pests and invasive species^20^,^21^ but is mostly confined to native tropical forest vegetation. The classification of the tribe has never been revised comprehensively, but taxonomic reviews have been published for the faunas of Australia and New Guinea^22^, Africa^23^, India^20^, China^24^,^25^, the Neotropical region^9^, and for the tropical Asian species originally described by C. F. Baker^26^. Recently, four new genera and several new species were reported from Australia, China, India and Thailand^27^,^28^,^29^. Species of the small tribe Balbillini are restricted to the Old World tropics^20^.

The previous morphology-based phylogeny of Evacanthinae did not include a large enough taxon sample to test the monophyly and relationships of the tribes included in the subfamily^9^. Only three species were included to represent the Evacanthini, and Balbillini and Pagaroniini were each represented by a single species. The only previous molecular phylogeny that included representatives of Evacanthinae is the 28S rDNA sequence-based phylogeny of Dietrich *et al*.^2^ subsequently augmented with morphological data^3^. This phylogeny placed the single included representative of Nirvaninae (*Australnirvana* [as *Nirvana*] *adelaideae* Evans) as sister to a clade comprising *Evacanthus* and two species of Pagaroniini, but with low branch support.

Recent molecular phylogenetic studies of leafhoppers and related groups have yielded additional gene regions informative of relationships among leafhopper genera and well-resolved phylogenetic estimates for tribes and genera within some other leafhopper subfamilies^4^,^6^,^30^,^31^.

The current study is the first comprehensive attempt to reconstruct the relationships within the subfamily Evacanthinae and test the classification in a phylogenetic framework. A comprehensive phylogenetic estimate will allow us to address the following questions. (1) Is Evacanthinae monophyletic and are the relationships recovered in Dietrich’s^9^ morphology-based phylogeny supported by molecular data? (2) Are the included tribes monophyletic and how are they related to each other? (3) What are the status and relationships of genera? (4) In which geographic regions did the subfamily and other monophyletic lineages originate? (5) What historical events and conditions contributed to shaping their current distribution? We also comment on and illustrate some of the remarkable morphological diversity within the subfamily.

## Results

### Phylogenetic analyses

Of the 81 taxa for which DNA extracts were available, nuclear genes 28S rDNA (*28S) D2* and *D9-D10* were successfully amplified for all taxa (see Supplementary Table S1), 73 for mitochondrial gene cytochrome c oxidase I (*COI*), 67 for Histone H3 (*H3*) and 66 for Wingless (*WG*) were successfully amplified. A new pair of primers was designed to amplify *H3* (see Supplementary Table S2) because of the low success rate of primers used in previous studies. In total, a matrix length of 2805 bp for the combined DNA sequences was obtained and used for the phylogenetic analyses. The taxon sample included all known genera of Balbillini (two in total) and Evacanthini (26 in total), 20 of the 35 known genera of Nirvanini and three of the four known genera of Pagaroniini. This sample provided broad representation of the world fauna, including all major biogeographic regions in which Evacanthinae are known to occur.

**Figure 1:**
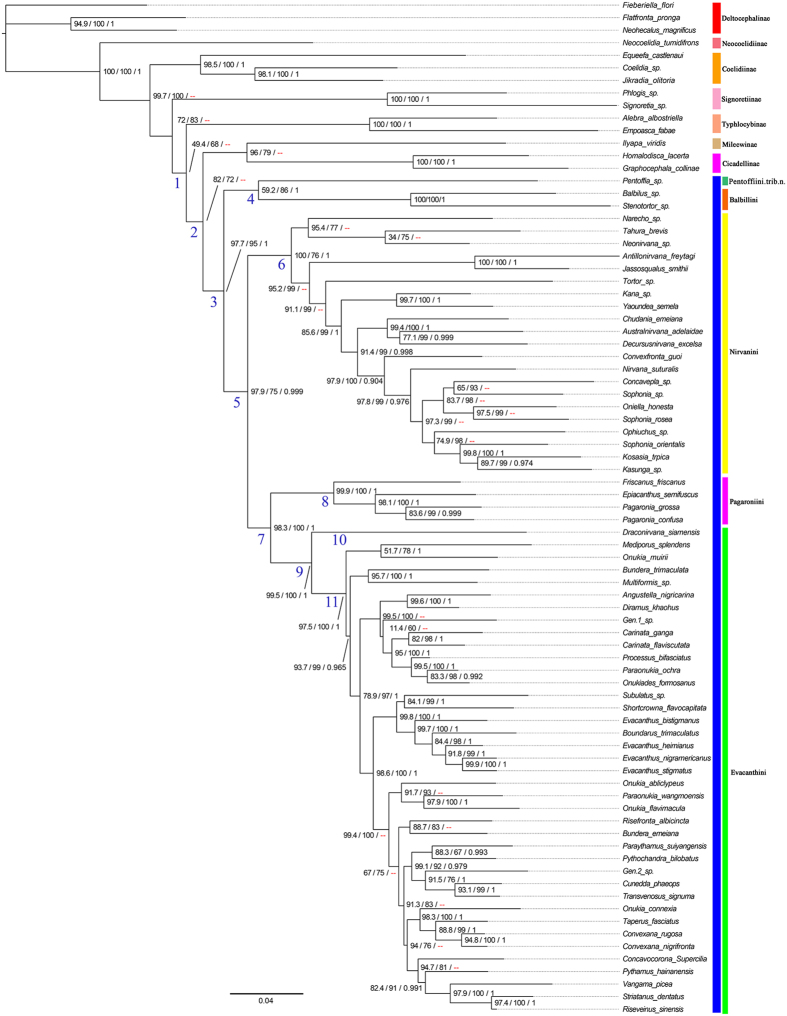
Maximum-likelihood (ML) tree estimated from the combined dataset. Major lineages are numbered and refer to text. At each node, values indicate ML support and Bayesian posterior probability (PP): SH-like approximate likelihood ratio test (SH-aLRT)/ultrafast bootstrap (UFB)/Bayesian PP values. Red “–” indicates clade not recovered in Bayesian analysis.

The phylogram from maximum likelihood (ML) analysis of combined data is shown in Fig. 1. The tree is well resolved and both SH-like approximate likelihood ratio test (SH-aLRT)^32^ and ultrafast bootstrap (UFB)^33^ scores indicate that most branches have moderate to high support. Only 11 nodes have SH-aLRT scores <80, and 22 nodes received UFB scores <95. The phylogram from Bayesian inference (BI) analysis of combined data can be found as Supplementary Fig. S1. Only seven nodes have posterior probability (PP) values <0.95. The ML and BI topologies are compatible, only differing in the poorly supported nodes. Bayesian PP values are shown on the ML phylogram (Fig. 1), and a red “–” indicates that the clade was not recovered in the Bayesian analysis. The phylogram from ML analysis of molecular data only can be found as Supplementary Fig. S2. The ML topologies based on molecular data only are highly similar to those obtained from the combined data, differing only in a few tip branches within Nirvanini and Evacanthini, and with lower branch support for several nodes. A list of morphological apomorphies for each resolved node on the tree shown in Fig. 1 is also given as Supplemental Text S2. The phylogram from Maximum parsimony (MP) of morphological data alone for the 81 included taxa can be found as Supplementary Fig. S3.

The analysis consistently recovered a clade (Fig. 1, clade 3) comprising Nirvanini, Balbillini, Evacanthini, Pagaroniini and *Pentoffia* Kramer with strong branch support, thus supporting the monophyly of Evacanthinae *sensu* Dietrich^9^. In contrast, branches subtending Evacanthinae received only moderate to low support, indicating that the relationship of Evacanthinae to other cicadellid subfamilies remains poorly resolved by our data.

Tribe Nirvanini was recovered as monophyletic with the exception of the genus *Draconirvana* Dietrich, which Dietrich^27^ described as morphologically intermediate between Evacanthini and Nirvanini but placed in the latter tribe. *Draconirvana* is here recovered as sister group to Evacanthini with strong support (Fig. 1). The other previously recognized tribes of Evacanthinae were recovered as monophyletic with strong support.

Tribe Pagaroniini (Fig. 1, clade 8) was recovered as the sister group of Evacanthini (plus *Draconirvana*, Fig. 1, clade 10) and together they form the sister group of Nirvanini (Fig. 1, clade 6). *Pentoffia* Kramer was the sister to Balbillini but with only moderate ML bootstrap support; together (Fig. 1, clade 4) they are sister to the remaining Evacanthinae (Fig. 1, clade 5).

Among genera for which more than one species was included, *Sophonia* Walker, *Evacanthus* Le Peletier & Serville, *Bundera* Distant, *Paraonukia* Ishihara and *Onukia* Matsumura were each not monophyletic.

The phylogeny resulting from Bayesian analysis (see Supplementary Fig. S2) was similar to the ML tree with the only differences occurring in some areas of the tree with low to moderate branch support in one or both analyses, e.g., relationships among some genera of Nirvanini and Evacanthini. Relationships among evacanthine tribes were identical and strongly supported by both analyses.

### Divergence times

**Figure 2:**
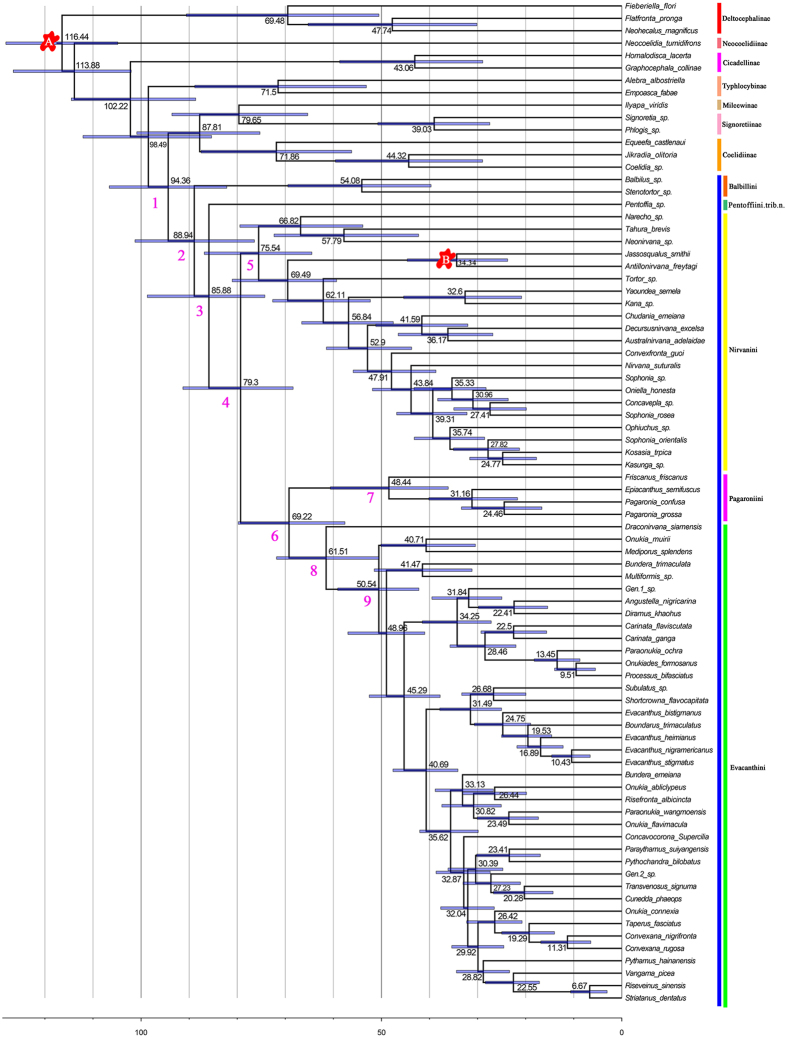
Maximum clade credibility tree from BEAST divergence time analysis. Stars with letters indicate calibration points. Major lineages are numbered and refer to text. Scale bar estimates age in millions of years. Each node is documented which its estimated age. Node bar represents 95% confidence interval (CI).

The divergence time analysis yielded the chronogram shown in Fig. 2. Divergence of Evacanthinae from other leafhoppers (Fig. 2, clade 1) was estimated to have occurred 94.36 Ma (82.2–106.6 Ma 95% confidence interval [CI]), and the crown age of Evacanthinae was estimated at 88.94 Ma (76.4–102.2 Ma 95% CI) (Fig. 2, clade 2). Divergence of Nirvanini from Evacanthini + Pagaroniini (Fig. 2, clade 4) was estimated to have occurred 79.3 Ma (68.4–91.3 Ma 95% CI), and the ages of the most recent common ancestors of Nirvanini, Evacanthini and Pagaroniini were 75.54 Ma (64.5–86.8 Ma 95% CI) (Fig. 2, clade 5), 61.51 Ma (50.5–71.8 Ma 95% CI) (Fig. 2, clade 8), 48.44 Ma (36.1–60.6 Ma 95% CI) (Fig. 2, clade 7) respectively.

### Biogeographical analyses

**Figure 3:**
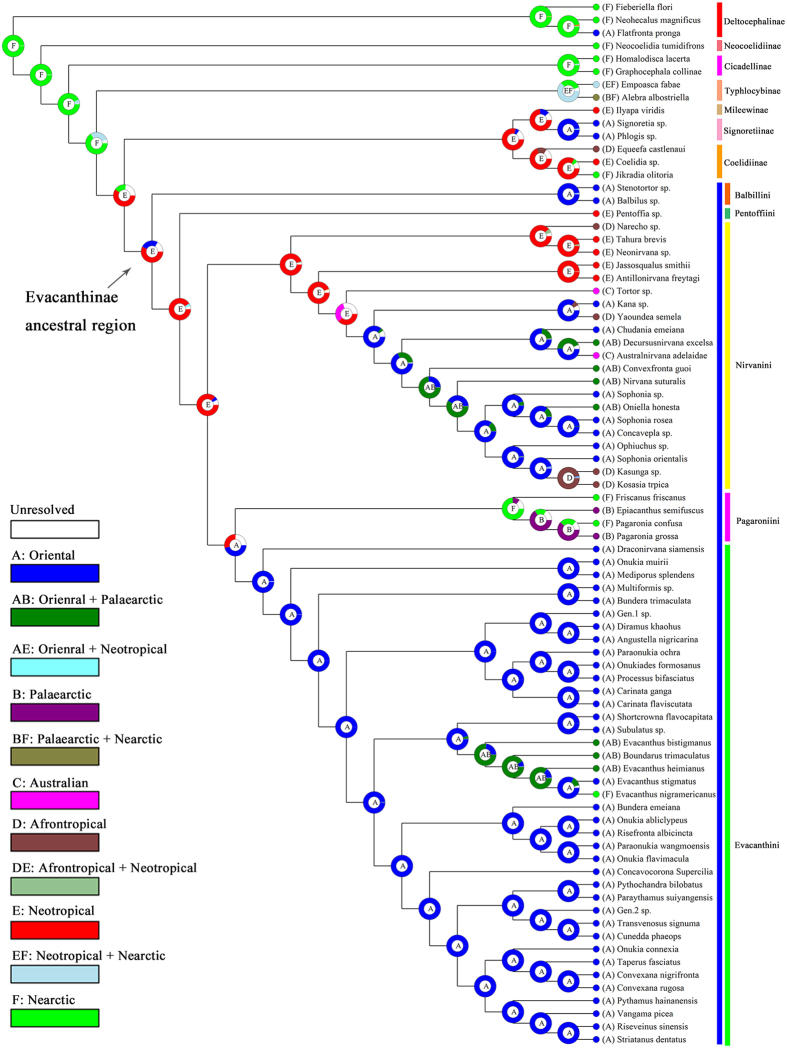
Graphical output from BBM analysis (exported from RASP) of ancestral distributions at each node of the phylogeny. Pie charts at each node show probabilities of alternative ancestral ranges, with the most likely state displayed in the centre. Coloured bars correspond to ancestral area reconstructions.

A phylogenetic tree showing the reconstruction of ancestral distribution ranges based on the BBM model is shown in Fig. 3. The ancestral region for Evacanthinae was somewhat equivocal but a Neotropical origin was slightly favored (57% probability) with Oriental less likely (26% probability). The Neotropical region was recovered as the most likely ancestral area for both Nirvanini (95% probability) and Nirvanini + Evacanthini + Pagaroniini (87% probability). The most likely ancestral area for Evacanthini was recovered as Oriental (99% probability). The ancestral region for Pagaroniini was recovered as Nearctic (75% probability).

## Discussion

Phylogenetic analyses of Evacanthinae based on combined molecular and morphological data recovered mostly well-resolved topologies with moderate to high support for most branches but poor resolution of relationships between Evacanthinae and representatives of other included leafhopper subfamilies. A previous analysis comprising representatives of most cicadellid subfamilies^2^ also failed to resolve the position of Evacanthinae among Cicadellinae, Mileewinae, Signoretiinae, Coelidiinae and Typhlocybinae. Thus, identification of the sister group of Evacanthinae must await further analyses of Cicadellidae as a whole that include characters more informative of relationships among major lineages of the family.

The present results provide strong support for the monophyly of Evacanthinae (*sensu lato*, Dietrich^9^) and its four previously included tribes, with the exception of the previous placement of *Draconirvana* (Fig. 1, clade 10) in Nirvanini^27^. Here we transfer *Draconirvana*, which was recovered as sister to the remaining Evacanthini to tribe Evacanthini (new placement). *Mediporus* Wang & Zhang, a more recently described genus of Evacanthini, was suggested to be related to *Draconirvana*^12^ and, in agreement with this suggestion, the present analysis placed *Mediporus* on the adjacent early-diverging lineage of Evacanthini. Prior to Dietrich’s morphology-based phylogenetic analysis^9^, Nirvaninae (with tribe Balbillini) had been treated as a separate subfamily and tribes Evacanthini and Pagaroniini had been included in Cicadellinae. Our results, based on analysis of the most taxonomically comprehensive and character-rich dataset to date for this leafhopper subfamily, support Dietrich’s morphology-based definitions of Evacanthinae and its included tribes, except for placement of *Draconirvana*, which keys to Nirvanini [and was placed in this tribe by Dietrich^27^] based on the incomplete median longitudinal carina of the frontoclypeus but, based on the present results, is more appropriately placed in Evacanthini (other genera of which have the median carina complete).

Kramer^34^ noted that *Pentoffia* is not close to any other genus of the Nirvaninae and only provisionally placed it in the subfamily (then treated as separate from Evacanthinae). Later, Hill^35^ provisionally placed the morphologically similar genus *Evanirvana* Hill in Evacanthini, but considered it to be intermediate between Evacanthini and Nirvaninae. A previous cladistic analysis of morphological characters placed *Pentoffia* as sister to the rest of Evacanthinae and, as a result, Dietrich^9^ treated both *Pentoffia* and *Evanirvana* as genera *incertae sedis* within Evacanthinae. Our analysis unequivocally placed *Pentoffia* within Evacanthinae but its position as sister group of Balbillini (Fig. 1, clade 4) received only moderate ML bootstrap support. Considering the unique combination of morphological characters shared by *Pentoffia* and *Evanirvana,* including the vestigial ocelli, medially carinate crown, and well-delineated forewing venation usually with three or more m-cu crossveins^9^, we propose to establish a new tribe Pentoffiini **trib.n.** within Evacanthinae based on these two genera (see below).

**Figure 4:**
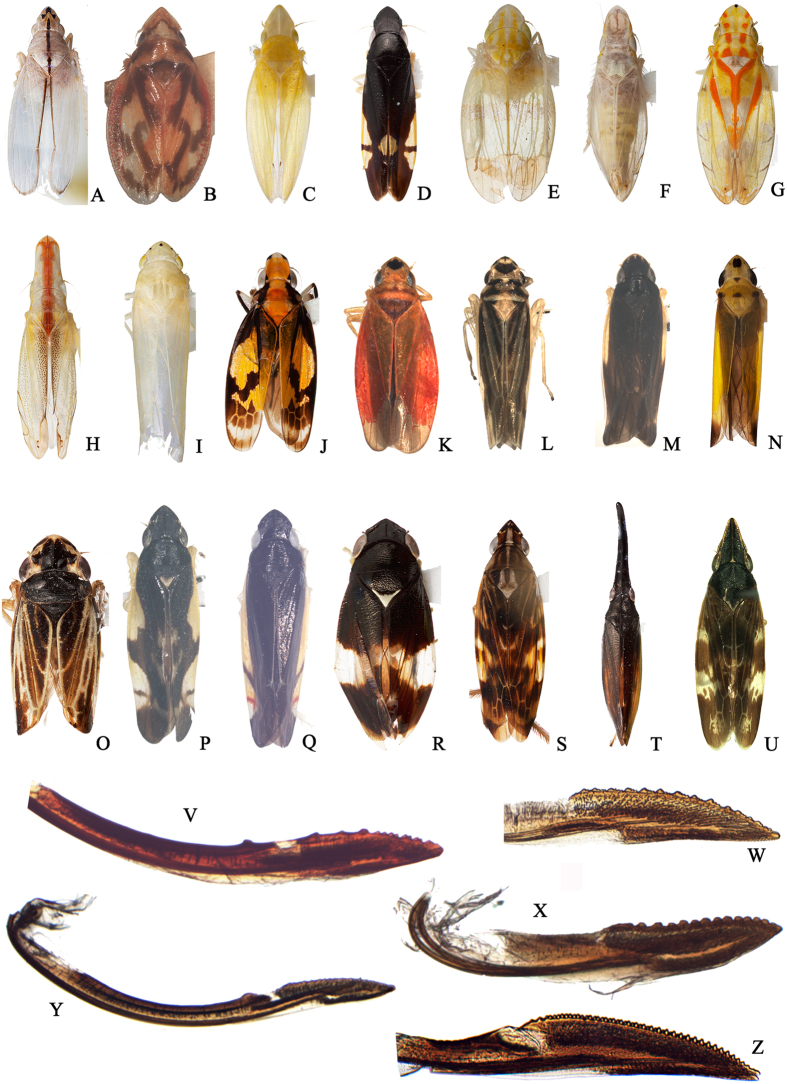
(**A**–**U)** Dorsal view of some species involved in current analysis: (**A**) *Pentoffia* sp.; (**B**) *Stenotortor* sp.; (**C**) *Narecho* sp.; (**D**) *Jassosqualus smithii*; (**E**) *Kana* sp.; (**F**) *Sophonia* sp.; (**G**) *Ophiuchus* sp.; (**H**), *Kasunga* sp.; (**I**) *Pagaronia grossa*; (**J**) *Draconirvana siamensis*; (**K**) *Onukia muirii*; (**L**) *Evacanthus bistigmanus*; (**M**) *Onukia connexia*; (**N**) *Onukiades formosanus*; (**O**) *Evacanthus nigramericanus*; (**P**) *Onukia flavimacula*; (**Q**) *Gen.*1 sp.; (**R**) *Risefronta albicincta*; (**S**) *Gen.*2 sp.; (**T**) *Vangama picea*; (**U**) *Riseveinus sinensis*. (**V**–**Z**) Female second valvulae: (**V**) *Pentoffia tridenta*; (**W**) *Afronirvana leptoclada*; (**X**) *Balbilus* sp.; (**Y**) *Pagaronia grossa*; (**Z**) *Transvenosus nigrodorsalis*.

The three species of the morphologically heterogeneous genus *Sophonia* Walker included in our dataset were not recovered as a monophyletic group. *Sophonia rosea* Li & Wang has a relatively short head, *Sophonia orientalis* (Matsumura) has a long head and convex crown, and *Sophonia* sp. (Fig. 4F), which resembles the type species of the genus, *S. rufitelum* Walker (known only from the female holotype), has a long head and concave crown. These results indicate that *Sophonia*, the largest genus of Nirvanini, needs to be redefined.

The results recovered the Oriental genus *Kana* Distant as sister to the African genus *Yaoundea* Linnavuori in agreement with the comment of Viraktamath Viraktamath and Wesley^20^ that these two genera may be synonymous. Recently, the only species of *Nirvana* recorded from Australia, *Nirvana adelaideae* Evans 1938, was removed to a new genus *Australnirvana*^29^. Our phylogenetic analyses provide support for this, indicating that the Australian species is more closely related to *Chudania* Distant and *Decursinirvana* Gao, Dai & Zhang than to *Nirvana* Kirkaldy.

Evacanthini includes the largest number of recognized genera among tribes of the subfamily but several of the included genera remain poorly characterized morphologically^12^. Our phylogenetic results indicate that *Evacanthus* Le Peletier & Serville, *Bundera* Distant, *Paraonukia* Ishihara and *Onukia* Matsumura are polyphyletic. Specifically, *Evacanthus* is paraphyletic with respect to *Boundarus* Li & Wang, with *B. trimaculatus* Li & Wang arising from within a clade comprising four *Evacanthus* species. *Onukia* is highly polyphyletic with the four included species placed in three separate, unrelated clades, although their positions varied somewhat in results from different analyses. The two included *Paraonukia* species were consistently placed on unrelated clades, one as sister to *Onukia flavimaculata* Li & Wang, the other as sister to *Onukiades formosanus* (Matsumura). The two included species of *Bundera* were also placed in distantly related clades, with one consistently placed as sister to *Multiformis* sp. and the other varying in position among results from different analyses. Wei *et al*.^13^ revised Baker’s species described in the Oriental leafhopper genus *Pythamus* Melichar, and placed them in a new genus *Pythochandra* Wei & Webb. In agreement with this work, the analysis indicated that *Pythochandra bilobatus* (Baker) and *Pythamus hainanensis* Wang, Dietrich & Zhang are only distantly related to each other. Representatives of two potential new genera, *Gen 1*.sp. (Fig. 4Q) and *Gen 2*.sp. (Fig. 4S) included in the analysis were placed on separate lineages only distantly related to established genera. These will be formally described in a separate paper.

Although our present results provide a strong foundation for classification of tribes and genera within Evacanthinae, the relationship of this group to other subfamilies of Cicadellidae remains unclear. In our results, the sister group of Evacanthinae was either a clade comprising Coelidiinae, Mileewinae and Signoretiinae (Bayesian results with 0.99 PP, see Supplementary Fig. S1), or Cicadellinae + Mileewinae (ML results with 82 UFB, Fig. 1). Previous results recovered Coelidiinae + Neocoelidiinae 9 or Mileewinae + Typhlocybinae^3^ as sister to Evacanthinae, all with only low to moderate branch support. Given the continued poor resolution of relationships between Evacanthinae and representatives of other included leafhopper subfamilies further analyses of Cicadellidae as a whole based on a large-scale molecular data (e.g., mitochondrial genomes and transcriptomes) are needed.

ML phylogram branch support assessed by UFB and SH-aLRT is generally considered to be significant if SH-aLRT > = 80 and UFB > = 95^36^. Based on these criteria, the current phylogram is reasonably well supported overall, although 11 nodes have SH-aLRT scores <80, and 22 nodes received UFB scores <95. Several factors contributed to this result: *28S D2, 28S D9-D10* and *H3* gene fragments have been shown to be informative for leafhopper phylogenetic analysis^2^,^4^,^37^; the DNA sequence dataset was reasonably complete, with missing data concentrated in a few relatively small gene regions; and the analysis of combined morphological and molecular data included large numbers of phylogenetically informative characters.

Because only 100 discrete morphological characters were included, the morphological data by themselves were not sufficient to yield a well resolved and supported phylogeny for Evacanthinae. Separate analysis of morphological data alone for the 81 included taxa yielded large numbers of equally parsimonious trees and the consensus tree (see Supplementary Fig. S3) had several large polytomies. Nevertheless, these analyses consistently recovered Balbillini, Evacanthini, Nirvanini and Pagaroniini as monophyletic and many relationships towards the tips of the tree were identical to those shown in Fig. 1. Areas of disagreement between the morphology-only trees and the trees based on molecular or combined data are attributable to poor resolution and relatively weak phylogenetic signal in one or both datasets. For example, *Gen.2* sp. (Fig. 4S) was recovered as the sister group of *Bundera emeiana* Li & Wang with relatively low branch support (20.5/32) in the molecular-only analyses (see Supplementary Fig. S2) but in the combined data analyses (Fig. 1) *Gen.2* sp. was recovered as sister group of *Cunedda phaeops* Distant + *Transvenosus signuma* Wang & Zhang with moderate branch support (91.5/76). *Gen.2* sp., *Cunedda phaeops* and *Transvenosus signuma* are morphologically similar in having the crown strongly concave between dorsally angulate median and lateral longitudinal keels; *Bundera emeiana* lacks this apparently derived morphological trait. Overall, addition of morphological data yielded a “total evidence” tree with higher branch support than was obtained for the tree based on molecular data alone (see Supplementary Fig. S2).

The divergence time estimates are very uncertain given the poor fossil record for Evacanthinae, and Cicadellidae in general. Only a single undoubted fossil belonging to Evacanthinae is known, from Oligo-Miocene Dominican amber^38^ and the group is, so far, conspicuously absent from the diverse and relatively well studied Eocene Baltic amber fauna, which has yielded representatives of several other modern leafhopper subfamilies^39^. There remains a major gap in the fossil record of leafhoppers between the oldest undoubted leafhoppers from lower Cretaceous rock fossils, most of which cannot be assigned to modern subfamilies, and the relatively diverse and well-preserved amber faunas of the Paleogene. Thus, although our molecular divergence time estimates suggest that the major lineages (tribes) of Evacanthinae diverged during the Cretaceous, there is no direct fossil evidence for this. Within Evacanthinae, divergences among major lineages and genera are suggested to have occurred mostly during the Paleogene, a plausible scenario given that most genera are restricted to the Oriental region, which was very geologically complex and dominated by tropical rainforest during that period.

The deepest clades are Balbillini and Pentoffiini (Figs 2 and 3). Balbillini is currently restricted to the Oriental and Afrotropical regions^23^, and Pentoffiini is restricted to the Neotropical region. Divergence of Evacanthinae from other leafhoppers (Fig. 2, clade 1) was estimated to have occurred 94.36 Ma (82.2–106.6 Ma 95% CI). So it seems likely that ancestral Evacanthinae were widespread in Gondwana before it completely divided, given the upper range of possible dates within the 95% confidence intervals. The earliest divergences in the subfamily (e.g., between Balbillini and Pentoffiini, and between Balbillini + Pentoffiini and the remaining lineages) may have occurred during the breakup of Gondwana, which is plausible given the range of possible dates within the 95% confidence intervals (76.4–102.2 Ma) (Fig. 2, clade 2) but they follow no clear Gondwanan vicariant sequence.

Inferred areas of origin, particularly for clades near the base of the tree, also need to be interpreted with caution because these may have been biased somewhat by the choice of outgroups, the unresolved relationship of Evacanthinae to other leafhoppers, and the in-group taxon sample. For example, only four pagaroniines (two Nearctic, two from Japan) are included in this study. Although the endemic western North American genus *Friscanus* Oman was sister to the remaining pagaroniines, inclusion of more species from Japan [which has a more species-rich fauna than that of North America^19^] may have increased the probability of a Palearctic area of origin for the tribe. Divergences among the four included pagaroniines are estimated to have occurred 48.44, 31.16 and 24.46 Ma (Fig. 2), and all three would have coincided with dispersal between Northeast Asia and the western Nearctic (Fig. 3). Given the existence of land connections and compatible flora and fauna in the Beringian region during the Paleogene (23–66 Ma)^40^,^41^, the present distribution of Pagaroniini is consistent with a Beringian dispersal hypothesis. One lineage of the leafhopper subfamily Iassinae (tribe Hyalojassini) was suggested to have a similar pattern of dispersal over higher latitudes from East Asia into North America at about the same time^4^.

Evacanthini includes one genus (*Evacanthus*) that occurs throughout the Holarctic region but is otherwise restricted to the Oriental region, particularly in southern China and the Indochinese Peninsula. The most likely ancestral area for Evacanthini was recovered as Oriental (99% probability) (Fig. 3), and given that the earliest diverging lineages of the tribe are species from Thailand (*Draconirvana siamensis* Dietrich and *Mediporus splendens* Wang & Zhang) and Malaysia (*Onukia muirii* Baker), it seems that Evacanthini originated in this region (Indochina Peninsula).

The biogeographic pattern of dispersal in Nirvanini appears to have been in the opposite direction to that observed in the iassine tribe Hyalojassini^4^. Nirvanini appears to have originated in the Neotropics and later dispersed into the Oriental region (and Africa and Australia). So it seems that exchange of leafhopper faunas between Oriental and Neotropical regions occurred in both directions during the Paleogene. It is interesting that the earliest diverging lineage of Nirvanini includes genera that mostly occur in South America (*Tahura, Neonirvana*) while the later diverging New World lineage includes species from Central America and the Caribbean (*Jassoqualus, Antillonirvana*), corroborating the hypothesis of dispersal out of South America and eventually into East Asia.

Also, according to our results, there were three separate colonizations of Africa and two separate colonizations of Australia by lineages of Nirvanini, all of which appear to have occurred well after the break-up of Gondwana and are, thus, best explained by intercontinental dispersal. Two divergence events between Oriental and Afrotropical nirvanine lineages are estimated to have occurred at about 27.82 and 32.6 Ma respectively (Figs 2 and 3). Although the transboreal tropical forest spanning the northern continental area that now separates Africa from tropical Asia during the late Paleocene and early Eocene [ca. 50–52 Ma^42^] would presumably have allowed overland dispersal between these two regions, the divergence dates between sister pairs of Oriental and Afrotropical nirvanines appear too young to be explained by such a scenario. Instead, the divergence dates are more consistent with the hypothesis of long-distance dispersal from South Asia across the Indian Ocean into Africa, possibly facilitated in part by expansion of tropical forest areas during cyclical thermal maxima in the Oligocene and Miocene or periods of low sea level during Oligocene glacial cycles^42^. Instances of long-distance, trans-oceanic dispersal have been documented in deltocephaline leafhoppers^5^,^43^. Although Nirvanini was the least well represented tribe in our taxon sample, with only ~57% of known genera represented, we believe that the geographic coverage of this sample was sufficiently representative of the tribe as a whole to provide a reasonable estimate of the biogeographic pattern. Nevertheless, discovery of additional fossil Evacanthinae and denser phylogenetic sampling of extant taxa will facilitate more robust tests of these historical biogeographic scenarios. New genera and species of Evacanthinae are also being discovered at a rapid pace, suggesting that the world fauna of this group remains very incompletely documented.

## Taxonomy

### Evacanthinae Metcalf

Evacanthinae Metcalf, 1939a: 247 (replacement for Euacanthinae Crumb, 1911a: 234).

Nirvaniidae (sic) Baker, 1923a: 353.

Pythamiinae (sic) Baker, 1915b: 193.

Notes. Dietrich^9^ redefined the Evacanthinae to include the tribes Nirvanini, Balbillini, Evacanthini and Pagaroniini and provided detailed descriptions and diagnoses of these taxa.

Key to tribes of Evacanthinae


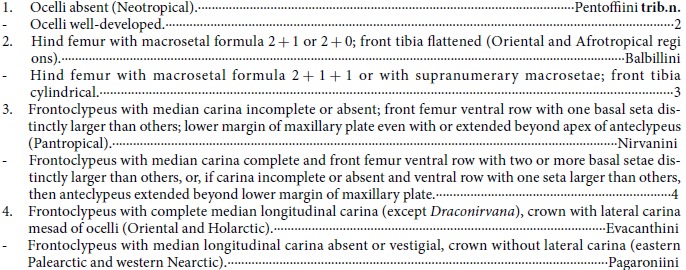


## Pentoffiini trib. n.

### Type genus: *Pentoffia* Kramer, 1964

Diagnosis. Medium-sized leafhoppers, usually pale, with or without dark markings. Head (Fig. 4A) moderately produced, expanded laterad anterad of eyes; coronal suture carinate and elevated towards apex; crown depressed medially, nearly horizontal, strongly elevated mesad of eyes, margin sharply carinate; ocelli absent; frontoclypeus in profile oblique, inflated, with median longitudinal carina dorsally; antennal ledges in anterior view narrow, flaplike; lorum very small and narrow, convex, well separated from margin of maxillary plate; gena broadly rounded, not emarginate below eye, mostly concealing large flaplike proepisternum. Forewing with well-delineated venation and usually with three or more m-cu crossveins. Hind femur with macrosetal formula 2 + 1 + 1. Female second valvula (Fig. 4V) with median dorsal tooth basad of toothed distal section, only slightly broadened preapically with irregular, asymmetrical dorsal teeth more prominent towards apex.

Notes. The new tribe Pentoffiini includes *Pentoffia* Kramer and *Evanirvana* Hill. Although the latter genus is known only from a few museum specimens and could not be included in the molecular dataset, it is very similar morphologically to *Pentoffia*, which was placed as sister to Balbillini in our phylogenetic estimates. As noted by Dietrich^9^, the main feature distinguishing *Evanirvana* from *Pentoffia* is the presence of a complete median keel on the crown of the former. Based on the phylogenetic results, Pentoffiini are most closely related to Balbillini but these two tribes are dramatically different morphologically, species of the latter being strongly flattened dorsoventrally with the ventral surface of the head horizontal. The dorsal teeth of the second valvulae of these two tribes are similar to each other in being blunt and irregular (Fig. 4V, X) compared to the acute and more evenly spaced and symmetrical teeth present in the other three tribes (Fig. 4W, Y, Z).

### *Draconirvana* Dietrch, placement n

In consideration of the depressed form and incomplete longitudinal carina on the face, traits that have traditionally been used to distinguish Nirvanini from related leafhoppers, Dietrich^27^ placed *Draconirvana* in Nirvanini but mentioned that it is morphologically intermediate between Evacanthini and Nirvanini. Our phylogenetic analysis confirmed its intermediate position but indicates that *Draconirvana* Dietrich is sister to a clade comprising the remaining genera of Evacanthini. Thus, we transfer this genus to Evacanthini. Presumably synapomorphic morphological features shared by *Draconirvana* and other Evacanthini include the strongly convex frontoclypeus, shallow antennal pits, keeled marginal carinae on the crown, pair of lateral oblique carinae mesad of the ocelli, and the tapered setae of the pecten of hind tarsomere I. *Mediporus*, a more recently described genus of Evacanthini, was suggested to be related to *Draconirvana*^12^. *Draconirvana* differs in having the crown strongly declivous, the longitudinal carina on the frontoclypeus incomplete, and the lower part of the face horizontal, as well as in the structure of the male genitalia.

## Material and methods

### Taxon sampling

Sixty-seven taxa were included representing all four evacanthine tribes plus genus *Pentoffia* Kramer (incertae sedis) and all regional faunas. Most included genera were represented by a single species but some larger and more morphologically heterogeneous genera were represented by multiple species to facilitate preliminary phylogenetic tests of the phylogenetic status of these genera. Fourteen additional taxa belonging to seven related subfamilies, Cicadellinae, Mileewinae, Signoretiinae, Coelidiinae, Typhlocybinae, Neocoelidiinae, Deltocephalinae, were selected as outgroups based on previous phylogenetic analyses that indicated possible relatedness to Evacanthinae^2^,^9^. Supplementary Table S1 shows the list of taxa and DNA sequences included in phylogenetic analyses.

### DNA extraction, amplification, sequencing

Field-collected leafhoppers were preserved in 95% ethanol. Upon processing, the ethanol was replaced and the samples were stored at −20 °C. Genomic DNA was extracted from the whole abdomens of single specimens by the DNeasy Tissue Kit (Qiagen, Inc.). Occasionally, DNA was also extracted from the entire leafhopper specimen if it was relatively small or old. We followed the manufacturer’s protocol, but with some modifications: the entire abdomen was incubated at 55 °C for 15–24 h, and DNA extractions were performed without destruction of the specimens, to allow for the subsequent examination of morphology and to preserve voucher material. Some DNA extractions were obtained from previous phylogenetic study of family Cicadellidae by Dietrich^2^ deposited at the Illinois Natural History Survey (INHS). Vouchers and DNA extracts newly included in this study are deposited at INHS and Northwest Agriculture and Forestry University, Yangling, Shaanxi, China.

Fragments of the genes *28S, WG* and *H3* and *COI* were amplified using primer pairs^2^,^44^,^45^,^46^,^47^,^48^ that can be found as Supplementary Table S2. These genes were selected based on their wide phylogenetic utility in published studies on leafhoppers. A previously published dataset of nearly complete 28S gene sequences for leafhoppers^2^ was screened and the *D2* and *D9-D10* regions were selected for amplification because they included the most characters potentially informative of relationships among Evacanthinae. Several *28S rDNA* and *COI* sequences were obtained from Genbank.

Fragments of *H3, WG* and *28S* were amplified by PCR in a total reaction volume of 25 μl using Taq Polymerase (Promega Corp.), held first for 3 min at 95 °C, then 30 cycles of 94 °C for 1 min, 55 °C for 1 min, and 72 °C for 2 min, then a final elongation step at 72 °C for 10 min, and held at 10 °C before being removed from the cycler. Primers used for amplification can be found as Supplementary Table S2. *COI* PCR products were amplified by primers LCO1490 and HCO2198 with the following thermal cycling protocol: 2 minutes at 95 °C; five cycles of 40 seconds at 94 °C, 40 seconds at 45 °C, and 1 minute at 72 °C; 35 cycles of 40 seconds at 94 °C, 40 seconds at 51 °C, and 1 minute at 72 °C; 10 minutes at 72 °C; and finally held at 10 °C^49^. When these primers were not successful, the primer cocktail C-tRWF_t1 enabled the amplification of the standard 658 bp barcode region, together with a short upstream sequence, in an additional 15% of the specimens^44^. PCR products were purified using Qiaquick PCR Purification Kit (Qiagen Inc.) or with GeneClean III Kit (MP Biomedicals). Both strands were sequenced using ABI Prism BigDye Terminator Kit version 3 (PE Applied Biosystems). Sequencing products were run on an ABI 3730XL capillary sequencer at the W.M. Keck Center for Comparative and Functional Genomics at the University of Illinois.

### Morphology

Morphological data consisted of 100 discrete binary and multistate characters of the exoskeleton (see Supplementary Text S1 and Table S3) treated as unordered and of equal weight. Missing or inapplicable states are indicated by “?” in the matrix and were treated as missing values in the analysis. Character states were scored using the same specimens from which DNA sequences were obtained, supplemented by one or more additional specimens in order to obtain data from both sexes. Morphological terminology follows previous work by Dietrich^9^,^27^.

### Alignment and phylogenetic analyses

Chromatograms were visualized in SEQUENCHER 5.1 (Gene Codes Corp.), and forward and reverse sequences were checked and assembled. Sequences were aligned with the MUSCLE application in MEGA 6^50^, and minor changes were subsequently made by hand. The *28S* data sets, which contained few or no ambiguously aligned positions, were manually adjusted as needed.

Combined ML analysis (*28S D2, 28S D9-D10, H3, WG, COI* and morphological data) and molecular-only ML analysis were conducted in IQtree v1.4.1^51^ using the best-fit substitution model automatically selected by the software according to the Bayesian information criterion scores and weights (BIC) with partitions. Separate data partitions were set up for each gene and morphology. An ultrafast bootstrap (UFB)^33^ with 10000 replicates and the SH-like approximate likelihood ratio test (SH-aLRT)^32^ were used in the analysis to assess branch support.

Combined BI analysis was conducted with MrBayes (v. 3.1.2)^52^ as implemented in CIPRES^53^. Separate data partitions were defined and unlinked for the *28S D2, 28S D9-D10, H3, WG, COI* and and morphology. The best-fit substitution model was selected by jModelTest 2.0 as implemented in CIPRES^53^. The SYM model was used for all molecular data partitions. The morphological dataset was run under the standard discrete model. Two runs with six chains each (three heated and three cold) were run for 20 million generations; the chains were sampled every 2,000 generations with default parameter values. Stationarity of the Markov Chain was determined as the point when sampled log-likelihood values plotted against generation time reached a stable mean equilibrium value; the first 25% of MCMC samples were discarded as ‘burn in’. Convergence of the Markov Chain and assessment of ‘burn in’ samples were determined using Tracer v1.4^54^. The morphological data were analysed under parsimony with the computer program TNT 1.5 (tree analysis using new technology)^55^ using a traditional search with 1000 replicates of random addition sequence followed by TBR (tree bisection and reconnection) branch swapping. Bremer supports using retain for suboptimal trees by 100 steps.

### Divergence time estimation

Divergence dates were inferred using the Bayesian relaxed clock uncorrelated lognormal method in BEAST^56^ as implemented in CIPRES^53^. The partitioned BEAST.xml input file was created with BEAUti v1.4.6^56^. A separate GTR + I + Γ model with four gamma categories was set and a Yule prior was set for branch lengths. Model parameters were unlinked across partitions. MCMC analyses were run for 20 million generations with parameters sampled every 2000 generations. The first 25% of the generations from each run were discarded as ‘burn in’. Convergence of the chains was checked using TRACER v1.4.1^54^. The searches achieved adequate mixing as assessed by the high effective sampling size (ESS) values for all parameters. Node ages and upper and lower bounds of the 95% highest posterior density (HPD) interval for divergence times were calculated using TreeAnnotator v1.5.4^56^ and visualized using FigTree v1.4.2. The 95% HPD represents the shortest interval that contains 95% of the sampled values from the posterior^56^.

### Calibrations

Because the fossil record of leafhoppers is extremely sparse and only a single representative of Evacanthinae is known from the fossil record, the oldest undoubted cicadellid fossil^57^,^58^ was used to constrain the maximum age of the root of the tree (~118 Ma). A normal distribution, which accounts for uncertainty in calibration estimates^59^ was used as a prior for the root node (A in Fig. 2), with a mean at 118 Ma (SD 6) and 97.5% confidence interval (CI) between 108 and 129.8 Ma. *Jassoqualus hispaniolensis* from Dominican amber^38^, the only known fossil representative of Evacanthinae, was used to calibrate the split between *Jassoqualus* and *Antillonirvana*. The age of Dominican amber, 15–20 Ma based on Foraminifera^60^, was used to calibrate this node (B in Fig. 2; minimum age). A log-normal prior distribution was specified (Logmean: 2.995) to allow the date to shift backwards in time because the divergence event likely occurred prior to the appearance of the fossil^59^.

### Biogeographical analyses

Ancestral areas were inferred by performing a Bayesian binary model (BBM) analysis implemented in RASP 3.2 using default parameter settings^61^. We chose this method because it provides probabilistic estimates of uncertainty in ancestral distributions, given the available data. We used a fixed (JC) model and equal among-site rate variation with the default chain parameters (50,000 cycles, 10 chains, with sampling every 100 generations and a temperature of 0.1) for the Bayesian analysis. Root distribution was set to Null and the maximum number of areas for each node was set to 2. Areas were defined based on known present-day distributions of extant taxa and coded as follows: A, Oriental; B, Palaearctic; C, Australian; D, Afrotropical; E, Neotropical; F, Nearctic. These broad geographic assignments were most appropriate for our data, which included a broad sample of the global fauna of Evacanthinae but did not include sufficiently dense sampling to infer finer-scale patterns within individual biogeographic realms.

## Additional Information

**How to cite this article:** Wang, Y. *et al*. Phylogeny and historical biogeography of leafhopper subfamily Evacanthinae (Hemiptera: Cicadellidae) based on morphological and molecular data. *Sci. Rep.* **7**, 45387; doi: 10.1038/srep45387 (2017).

**Publisher's note:** Springer Nature remains neutral with regard to jurisdictional claims in published maps and institutional affiliations.

## Supplementary Material

Supplementary Information
