# Supplementary material for: Phylogeny and historical biogeography of leafhopper subfamily Evacanthinae (Hemiptera: Cicadellidae) based on morphological and molecular data
Source: Sci Rep. 2017 Apr 3;7:45387. doi: 10.1038/srep45387 (PMC5377372; doi:10.1038/srep45387)
Supplement: Supplementary Information [file srep45387-s1.pdf]

Phylogeny and historical biogeography of leafhopper subfamily Evacanthinae (Hemiptera: Cicadellidae) based on morphological and molecular data

YANG WANG[1], CHRISTOPHER H. DIETRICH[2] & YALIN ZHANG[1, *]

[1]*Key Laboratory of Plant Protection Resources and Pest Management, Ministry of Education, Entomological Museum, Northwest A&F University, Yangling, Shaanxi 712100, China*
[2]*Illinois Natural History Survey, Prairie Research Institute, University of Illinois, 1816 S Oak St., Champaign, Illinois 61820, USA*
[*]*Corresponding authors. E-mail: yalinzh@nwafu.edu.cn*

Supplementary Text S1. Characters and states for the analysis in this study.

Colour

1. Dorsal coloration: (0) mostly pale green, orange, yellow or white; (1) mostly dark brown or black. Taxa scored as state 0 often vary among the colours indicated depending on the state of preservation of the specimen.

2. Body shape: (0) flat; (1) cylindrical.

Head

3. Length of head: (0) shorter than pronotum; (1) about same as pronotum; (2) longer than pronotum; (3) equal to or longer than pronotum and scutellum together.
4. Coronal suture: (0) not extended to crown apex; (1) extended to apex; (2) with carina; (3) absent.
5. Median longitudinal carina: (0) weak; (1) distinct; (2) strongly keeled; (3)incomplete; (4) absent.
6. Crown surface: (0) flat or convex; (1) weakly concave; (2) strongly concave.
7. Crown anterior margin shape in dorsal view: (0) rounded; (1) triangularly produced but not elongate; (2) elongate.
8. Head shape: (0) not depressed; (1) strongly dorsoventrally flattened.
9. Crown lateral carina presence: (0) absent; (1) present.
10. Crown lateral carina development: (0) incomplete; (1) complete; (2) strongly keeled.
11. Marginal carina: (0) absent; (1) present.
12. Marginal carina: (0) weak; (1) distinct; (2) strongly keeled.
13. Latero-ocellar carina: (0) absent; (1) present.
14. Lateral and marginal carina: (0) fused in the front of ocellus; (1) not fused.

15. Submarginal carina: (0) absent; (1) present.
16. Transverse carina: (0) absent; (1) present.
17. Additional complete carina between latero-ocellar carina and lateral carina: (0) absent; (1) present.
18. Pair gibbosities on basal half of crown: (0) absent; (1) present at basal half of head; (2) present medially each side of carina.
19. Crown texture: (0) smooth or finely granulose; (1) rugulose; (2) coarsely rugose.
20. Crown lateral emargination: (0) absent; (1) present.
21. Ocelli development: (0) vestigial; (1) well developed but small; (2) large.
22. Ocelli position: (0) on crown distant from margin; (1) on crown near margin; (2) on or just below margin.
23. Ocelli position: (0) very close to eye (the distance less than 1/4 of length between eye and apex of crown); (1) equidistant from eye and apex of crown; (2) close to eye (the distance about 1/4-1/3 of length between eye and apex of crown).
24. Distance between eyes: (0) more than 1/2 width of pronotum; (1) equal to or narrower than 1/2 width of pronotum.
25. Antennal ledge: (0) absent or greatly reduced; (1) well developed, oblique; (2) well developed, horizontal.
26. Antennal pit: (0) near anteroventral corner of eye; (1) near midheight of eye; (2) near anterodorsal corner of eye.
27. Flagellum: (0) short; (1) more than 1/2 body length.
28. Lateral frontal sutures: (0) absent or invisible externally; (1) extended to crown margin; (2) extended onto crown.
29. Frontoclypeus median longitudinal carina: (0) absent; (1) present dorsally; (2) extended from dorsal margin to clypeal suture.
30. Frontoclypeus oblique lateral carina: (0) absent; (1) present, but weak; (2) strong.
31. Frontoclypeus shape: (0) evenly convex; (1) flattened medially; (2) concave.
32. Lorum size: (0) small, narrower than clypellus; (1) large, wider than clypellus; (2) greatly enlarged.
33. Lorum distance from maxillary plate margin: (0) extended to margin; (1) well separated from margin.
34. Genal margin: (0) narrow, broadly emarginate; (1) wide, rounded; (2) angulately produced.
35. Clypellus shape: (0) tapered distally, convex; (1) parallel-sided or broadened distally, flattened.
36. Clypellus apex: (0) extended beyond maxillary plate margin; (1) even with maxillary plate margin; (2) not extended to maxillary plate margin.
37. Rostrum: (0) not surpassing front trochanters; (1) surpassing front trochanters; (2) extended to or beyond hind coxae.

Thorax

Pronotum

38. Length: (0) not extended to scutellum; (1) extended to scutellum.
39. Setigerous tubercles: (0) absent; (1) present.
40. Distinct foveae: (0) absent; (1) present.
41. Lateral carina position: (0) even with eye (or absent); (1) below eye.

Forewing

42. Venation: (0) weak, or only distinct on distal part; (1) normal; (2) strong, raised.
43. Vein R: (0) free; (1) fused with costal vein in the middle (Fig. 5H).
44. Vein R: (0) with 2 branches; (1) with 3 branches; (2) with more than 3 branches.
45. Crossvein s: (0) absent; (1) present.
46. Crossvein r-m1: (0) absent; (1) present.
47. R2 +3: (0) extended obliquely distad; (1) reflexed.
48. Crossvein m-cu2: (0) absent; (1) present; (2) with supranumerary crossveins between m-cu1 and m-cu3. State 2 is considered a modified form of state 1.
49. Apical cell 3: (0) >2 longer than wide; (1) only slightly, if any, longer than wide.
50. Inner apical cell: (0) short, tapered; (1) elongate, parallel-sided; (2) elongate, wider than other cells.
51. Cu: (0) connected to submarginal vein at claval apex; (1) connected to submarginal vein well distad of claval apex.
52. Appendix: (0) absent; (1) restricted to anal margin; (2) extended around wing apex.
53. False veins (costal maculae): (0) absent; (1) 1 present; (2) 2 present; (3) 3 present.
54. Claval veins: (0) separate; (1) fused for a short distance; (2) absent.

Hindwing

55. R2 +3: (0) absent; (1) obsolete distally; (2) complete.
56. R4 +5 and M1 +2: (0) free, with crossvein; (1) confluent.
57. Crossvein m-cu: (0) long, oblique; (1) short, straight; (2) absent, median vein fused with cubital vein for a short distance.

Front leg

Femur

58. AM1: (0) near middle of anterior surface; (1) on or near ventral margin.
59. Intercalary row: (0) setae of similar size; (1) 2–4 basal setae distinctly larger than others; (2) 1 basal seta distinctly enlarged.

Tibia

60. Shape: (0) cylindrical; (1) flattened dorsally.

Middle leg

61. Femur: (0) width subequal to front femur; (1) distinctly wider than front femur.

Hind leg

62. Femur length: (0) extended to proepimeron (lateral pronotal lobe) at rest; (1) not reaching proepimeron at rest.
63. Femur macrosetae: (0) 2 + 2 + 1; (1) 2 + 1 + 1; (2) 2 + 1; (3) 2 + 0.
64. Tibia row PD, basal seta: (0) even with or slightly distad of basal AD seta; (1) even with second seta of AD.
65. Tibia row AV: (0) extended nearly entire length; (1) restricted to distal 1/2.
66. Tarsus: (0) normal; (1) greatly elongate.
67. Tarsomere I pecten: (0) with 5 or more platellae; (1) with 3–4 platellae; (2) with 2 platellae; (3) without platellae (apex tapered).
68. Tarsomere I pecten platellae: (0) normal; (1) setiform.

Male pygofer

69. Macrosetae: (0) absent; (1) scattered; (2) in distinct group.
70. Dorsal process: (0) absent; (1) present.
71. Ventral process: (0) absent; (1) toothlike; (2) elongate, inornate; (3) elongate, stocky or branched.
72. Positon of ventral process: (0) basal; (1) distal.
73. Segment X shape: (0) semicylindrical; (1) cylindrical.
74. Anal hooks (basolateral processes of segment X) : (0) absent; (1) present.
75. Segment X posterolateral processes: (0) absent; (1) present.
76. Valve: (0) free; (1) fused to pygofer.
77. Subgenital plates: (0) free; (1) fused to valve.
78. Subgenital plates: (0) free; (1) partially fused to each other.
79. Subgenital plate macrosetae: (0) absent; (1) numerous, scattered; (2) numerous, uniseriate; (3) 6 or fewer.
80. Subgenital plate shape: (0) broad basally with apex attenuate; (1) triangular, depressed; (2) ligulate; (3) boatlike.

81. Subgenital plate apex: (0) not round and expanded; (1) rounded and expanded.
82. Connective: (0) Y shaped with arms only slightly divergent; (1) nearly T-shaped (arms strongly divergent); (2) X shape.
83. Stem and arm of connective: (0) arm shorter than 1/4 length of stem; (1) arm shorter than stem, but longer than 1/4 length of stem (2) stem almost equal with arm or longer.
84. Connective: (0) without median anterior lobe; (1) with median anterior lobe.
85. Style apodeme: (0) elongate; (1) short.
86. Style preapical lobe: (0) absent; (1) short, rounded; (2) large, acute.
87. Style apophysis: (0) without preapical tooth; (1) with preapical tooth; (2) preapical tooth on heellike extension.
88. Apex of style apophysis: (0) poorly developed; (1) well developed, about 1/3 length of style; (2) well developed, about 1/2 length of style or longer.
89. Aedeagus, ventral process: (0) absent; (1) poorly developed, odontoid; (2) well developed, salient; (3) well developed, elongate or branched.
90. Aedeagus, paired dorsal atrial processes: (0) absent; (1) present.
91. Aedeagus dorsal apodeme: (0) absent or weakly sclerotized; (1) divided into pair of lateral arms; (2) arms fused dorsomedially; (3) with single compressed dorsomedial process.
92. Aedeagus, paired distal processes: (0) absent; (1) simple; (2) branched.
93. Aedeagal shaft: (0) recurved; (1) straight; (2) arcuate.
94. Aedeagal shape in lateral view: (0) similar to *Cunedda phaeopes* (shaft recurved dorsally, apodeme arising from atrium and extended dorsolaterad; ventral apophysis strong raised); (1) other types.

Female

95. First valvulae sculpturing: (0) strigate; (1) concatenate/areolate; (2) granulose.

Second valvulae

96. Middorsal cleft: (0) absent; (1) present.
97. Middorsal tooth: (0) absent; (1) simple; (2) coelidiinelike with pair of lateral teeth just basad of medial tooth.
98. Dorsal serrations: (0) symmetrical, simple; (1) symmetrical, with smaller teeth between larger ones; (2) asymmetrical, irregular.
99. Shape: (0) not strongly sinuate; (1) strongly sinuate.
100. Toothed blade length: (0) less than 1/3 total length; (1) more than 2/3 total length

Supplementary Text S2.. Morphological apomorphies.

Numbered nodes on the following tree diagram correspond to those in the list of apomorphies given below. ACCTRAN character optimization was used. The following tree is ML tree estimated from the combined dataset, the same one as shown in Figure 1.

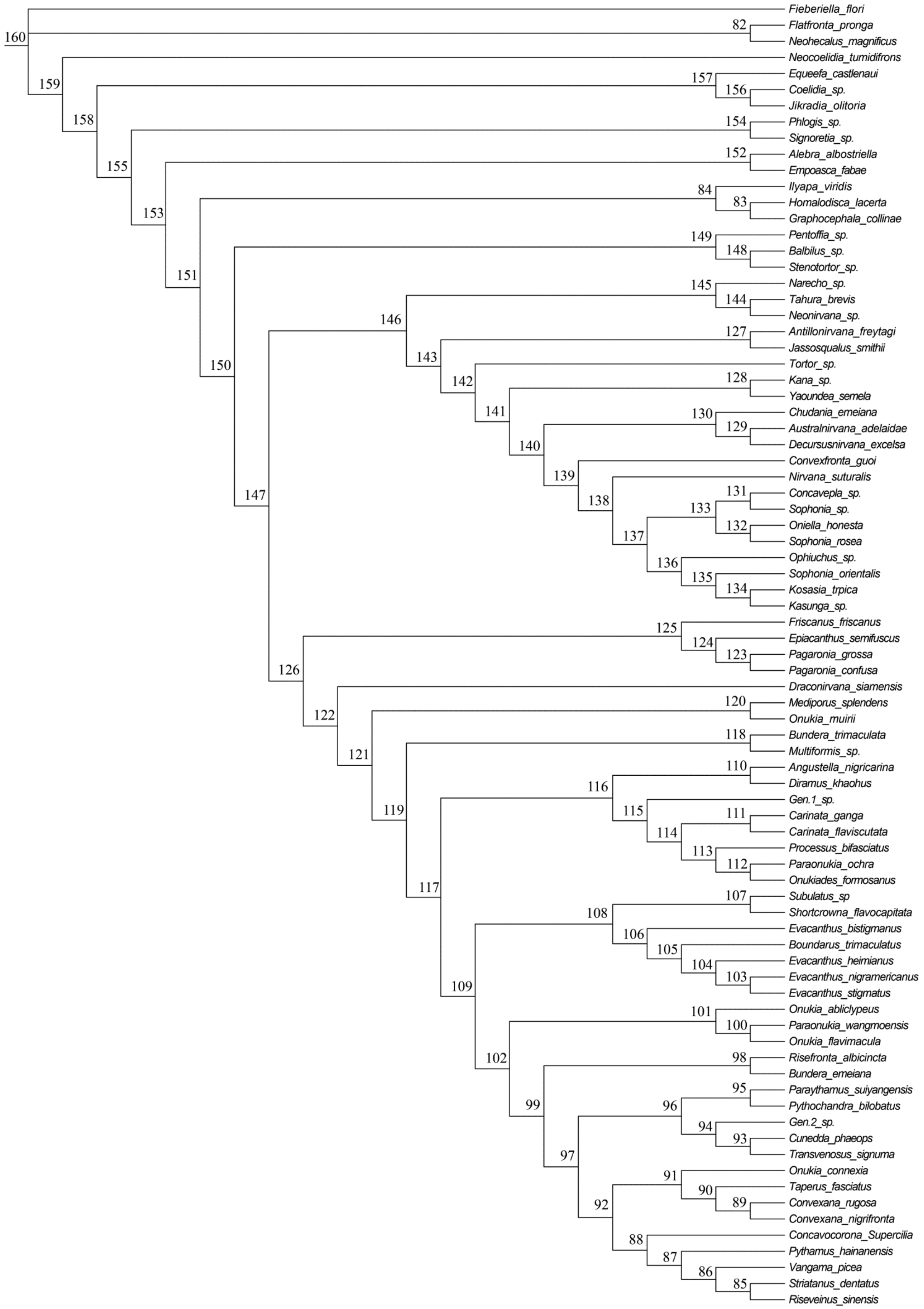

Apomorphy lists:

Branch	Character	Steps	CI	Change
node_160 --> Fieberiella flori	44	1	0.133	1 --> 2
	46	1	0.167	0 --> 1
	48	1	0.091	0 --> 1
	51	1	0.200	0 --> 1
	56	1	0.077	0 --> 1
	71	1	0.094	0 --> 3
	74	1	0.111	0 ==> 1
	88	1	0.074	0 ==> 1
	91	1	0.125	0 --> 3
node_160 --> node_82	36	1	0.125	0 ==> 1
	72	1	0.091	0 --> 1
	79	1	0.136	0 --> 2
	86	1	0.087	0 --> 1
node_82 --> Flatfronta pronga	12	1	0.143	1 ==> 0
	26	1	0.286	1 ==> 2
	31	1	0.300	0 ==> 2
	33	1	0.500	1 ==> 0
	48	1	0.091	0 --> 1
	50	1	0.154	1 ==> 2
	67	1	0.273	0 ==> 1
	71	1	0.094	0 --> 3
	85	1	0.250	0 --> 1
	90	1	0.067	1 ==> 0
node_82 --> Neohecalus magnificus	3	1	0.130	0 ==> 2
	30	1	0.167	0 ==> 1
	44	1	0.133	1 --> 0
	46	1	0.167	0 --> 1
	53	1	0.200	0 ==> 1
	69	1	0.154	1 ==> 2
	86	1	0.087	1 --> 2
	91	1	0.125	0 --> 3
	92	1	0.087	0 --> 1
	98	1	0.286	0 ==> 2
	100	1	0.333	0 ==> 1
node_160 --> node_159	2	1	0.200	0 ==> 1
	4	1	0.167	0 ==> 2
	7	1	0.091	1 ==> 0
	11	1	0.200	1 ==> 0
	23	1	0.182	0 --> 1
	25	1	0.182	0 --> 2
	34	1	0.250	2 --> 0
	37	1	0.250	0 ==> 1
	41	1	0.167	0 --> 1
	45	1	0.125	0 --> 1
	49	1	0.143	1 ==> 0

	58	1	0.333	1 ==> 0
	69	1	0.154	1 ==> 0
	70	1	0.125	0 --> 1
	80	1	1.000	1 --> 2
	83	1	0.083	2 ==> 1
	85	1	0.250	0 --> 1
	92	1	0.087	0 --> 1
node_159 --> node_158	30	1	0.167	0 ==> 1
	57	1	0.333	1 --> 0
	67	1	0.273	0 ==> 1
	76	1	0.333	0 --> 1
node_158 --> node_155	32	1	0.500	1 --> 0
	35	1	1.000	1 ==> 0
	41	1	0.167	1 --> 0
	61	1	0.500	1 --> 0
	63	1	0.600	0 --> 1
	70	1	0.125	1 --> 0
	91	1	0.125	0 --> 1
node_155 --> node_153	4	1	0.167	2 --> 1
	10	1	0.182	0 --> 1
	34	1	0.250	0 --> 1
	45	1	0.125	1 --> 0
	79	1	0.136	0 --> 1
	82	1	0.118	0 ==> 1
node_153 --> node_151	3	1	0.130	0 --> 1
	7	1	0.091	0 --> 1
	22	1	0.400	2 --> 0
	23	1	0.182	1 --> 2
	25	1	0.182	2 ==> 1
	36	1	0.125	0 --> 1
	37	1	0.250	1 --> 0
	48	1	0.091	0 --> 1
	61	1	0.500	0 --> 1
	64	1	0.250	0 ==> 1
	69	1	0.154	0 --> 2
	84	1	0.083	0 --> 1
	85	1	0.250	1 ==> 0
node_151 --> node_84	28	1	0.250	1 ==> 2
	31	1	0.300	0 --> 1
	34	1	0.250	1 --> 0
	45	1	0.125	0 --> 1
	46	1	0.167	0 ==> 1
	50	1	0.154	1 --> 2
	62	1	0.250	0 ==> 1
	68	1	0.200	0 --> 1
	100	1	0.333	0 ==> 1
node_84 --> node_83	21	1	0.333	1 ==> 2
	36	1	0.125	1 --> 0
	37	1	0.250	0 --> 1

	41	1	0.167	0 --> 1
	52	1	0.400	1 ==> 2
	55	1	0.500	2 --> 0
	69	1	0.154	2 --> 0
	73	1	0.333	0 ==> 1
	76	1	0.333	1 ==> 0
	79	1	0.136	1 --> 0
	80	1	1.000	2 ==> 0
	82	1	0.118	1 ==> 0
	84	1	0.083	1 --> 0
node_83 --> Homalodisca lacerta	1	1	0.071	0 ==> 1
	6	1	0.111	0 ==> 1
	25	1	0.182	1 ==> 2
	54	1	0.667	0 ==> 1
	55	1	0.500	0 --> 1
	63	1	0.600	1 ==> 2
	67	1	0.273	1 ==> 0
	83	1	0.083	1 ==> 2
	93	1	0.333	0 ==> 1
node_83 --> Graphocephala collinae	7	1	0.091	1 --> 0
	31	1	0.300	1 --> 0
	50	1	0.154	2 --> 1
	68	1	0.200	1 --> 0
	69	1	0.154	0 --> 1
	79	1	0.136	0 --> 2
	92	1	0.087	1 ==> 0
node_84 --> Ilyapa viridis	2	1	0.200	1 ==> 0
	3	1	0.130	1 --> 0
	4	1	0.167	1 --> 0
	26	1	0.286	1 ==> 2
	44	1	0.133	1 --> 2
	47	1	0.200	0 ==> 1
	48	1	0.091	1 --> 2
	49	1	0.143	0 ==> 1
	58	1	0.333	0 ==> 1
	71	1	0.094	0 --> 2
	75	1	0.500	0 ==> 1
	86	1	0.087	0 ==> 2
	90	1	0.067	1 ==> 0
	91	1	0.125	1 --> 3
	98	1	0.286	0 ==> 2
node_151 --> node_150	4	1	0.167	1 --> 2
	11	1	0.200	0 ==> 1
	22	1	0.400	0 --> 1
	29	1	0.286	0 --> 1
	57	1	0.333	0 --> 1
	59	1	0.154	0 --> 2
	96	1	0.200	0 --> 1
node_150 --> node_147	3	1	0.130	1 --> 2

	48	1	0.091	1 --> 0
	71	1	0.094	0 --> 3
	91	1	0.125	1 --> 2
node_147 --> node_126	34	1	0.250	1 --> 0
	36	1	0.125	1 --> 0
	37	1	0.250	0 --> 1
	41	1	0.167	0 --> 1
	59	1	0.154	2 --> 1
	69	1	0.154	2 --> 0
	86	1	0.087	0 --> 1
node_126 --> node_122	5	1	0.250	4 ==> 1
	6	1	0.111	0 --> 2
	9	1	0.333	0 ==> 1
	23	1	0.182	2 ==> 1
	25	1	0.182	1 ==> 0
	68	1	0.200	0 ==> 1
	71	1	0.094	3 --> 0
	82	1	0.118	1 ==> 0
	84	1	0.083	1 ==> 0
	95	1	0.250	1 ==> 0
node_122 --> node_121	17	1	0.100	0 ==> 1
	29	1	0.286	1 --> 2
	87	1	0.095	0 ==> 1
	89	1	0.111	0 ==> 2
	91	1	0.125	2 --> 1
	92	1	0.087	1 ==> 0
node_121 --> node_119	1	1	0.071	0 ==> 1
	3	1	0.130	2 --> 1
	6	1	0.111	2 --> 1
	13	1	0.143	0 ==> 1
node_117 --> node_109	6	1	0.111	1 --> 2
	18	1	0.200	0 --> 1
	87	1	0.095	1 --> 0
	88	1	0.074	0 --> 2
node_109 --> node_102	10	1	0.182	1 --> 2
	94	1	0.167	1 ==> 0
node_102 --> node_99	17	1	0.100	1 --> 0
	87	1	0.095	0 --> 2
node_99 --> node_97	7	1	0.091	1 --> 2
	18	1	0.200	1 --> 0
node_97 --> node_92	19	1	0.105	0 --> 1
	94	1	0.167	0 --> 1
node_92 --> node_88	3	1	0.130	1 --> 0
	12	1	0.143	1 ==> 2
	13	1	0.143	1 ==> 0
	14	1	0.500	1 ==> 0
	16	1	0.167	0 ==> 1
	19	1	0.105	1 --> 2
	23	1	0.182	1 ==> 2

	59	1	0.154	1 --> 0
node_88 --> node_87	3	1	0.130	0 --> 2
	5	1	0.250	1 ==> 2
	88	1	0.074	2 --> 1
	89	1	0.111	2 --> 0
node_87 --> node_86	3	1	0.130	2 --> 3
	18	1	0.200	0 --> 2
	54	1	0.667	0 ==> 1
	82	1	0.118	0 ==> 1
	89	1	0.111	0 --> 3
node_86 --> node_85	16	1	0.167	1 ==> 0
	42	1	0.250	1 ==> 2
	83	1	0.083	1 ==> 0
	84	1	0.083	0 ==> 1
node_85 --> Striatanus dentatus	71	1	0.094	0 ==> 3
	86	1	0.087	1 ==> 0
	88	1	0.074	1 --> 2
	89	1	0.111	3 ==> 1
	92	1	0.087	0 ==> 1
node_86 --> Vangama picea	74	1	0.111	0 ==> 1
	75	1	0.500	0 ==> 1
	87	1	0.095	2 ==> 0
	88	1	0.074	1 ==> 0
	90	1	0.067	1 ==> 0
	91	1	0.125	1 ==> 0
node_87 --> Pythamus hainanensis	36	1	0.125	0 ==> 1
	59	1	0.154	0 --> 1
node_88 --> Concavocorona Supercilia	7	1	0.091	2 --> 1
	18	1	0.200	0 --> 1
	71	1	0.094	0 ==> 2
	94	1	0.167	1 --> 0
node_92 --> node_91	6	1	0.111	2 --> 0
	10	1	0.182	2 ==> 1
	17	1	0.100	0 --> 1
	79	1	0.136	1 ==> 2
	83	1	0.083	1 --> 0
node_90 --> Taperus fasciatus	23	1	0.182	1 ==> 2
	69	1	0.154	0 ==> 2
	83	1	0.083	0 --> 1
node_90 --> node_89	6	1	0.111	0 ==> 1
	82	1	0.118	0 ==> 1
	84	1	0.083	0 ==> 1
node_89 --> Convexana rugosa	17	1	0.100	1 ==> 0
	88	1	0.074	2 ==> 1
	89	1	0.111	2 ==> 1
node_89 --> Convexana nigrifronta	7	1	0.091	2 --> 1
	48	1	0.091	0 ==> 1
node_91 --> Onukia connexia	7	1	0.091	2 --> 1
	30	1	0.167	1 ==> 0

	89	1	0.111	2 ==> 0
	92	1	0.087	0 ==> 1
node_97 --> node_96	5	1	0.250	1 ==> 2
	18	1	0.200	0 --> 2
	42	1	0.250	1 --> 2
node_96 --> node_94	12	1	0.143	1 ==> 0
node_94 --> node_93	13	1	0.143	1 ==> 0
	17	1	0.100	0 --> 1
node_93 --> Cunedda phaeops	42	1	0.250	2 --> 1
node_93 --> Transvenosus signuma	71	1	0.094	0 ==> 2
node_94 --> Gen.2 sp.n.	37	1	0.250	1 ==> 2
	59	1	0.154	1 ==> 0
	83	1	0.083	1 ==> 0
node_95 --> Paraythamus suiyangensis	17	1	0.100	0 --> 1
	48	1	0.091	0 ==> 1
	59	1	0.154	1 ==> 0
	79	1	0.136	1 ==> 2
	87	1	0.095	2 ==> 1
	88	1	0.074	2 ==> 1
	89	1	0.111	2 ==> 0
	91	1	0.125	1 ==> 2
	92	1	0.087	0 ==> 1
	94	1	0.167	0 ==> 1
node_95 --> Pythochandra bilobatus	19	1	0.105	0 ==> 1
	42	1	0.250	2 --> 1
	62	1	0.250	0 ==> 1
	71	1	0.094	0 ==> 3
	82	1	0.118	0 ==> 1
	83	1	0.083	1 ==> 0
node_99 --> node_98	6	1	0.111	2 --> 0
	16	1	0.167	0 ==> 1
node_98 --> Risefronta albicincta	69	1	0.154	0 ==> 2
	71	1	0.094	0 ==> 2
	83	1	0.083	1 ==> 0
node_98 --> Bundera emeiana	10	1	0.182	2 ==> 1
	56	1	0.077	0 ==> 1
	86	1	0.087	1 ==> 0
node_102 --> node_101	13	1	0.143	1 ==> 0
	70	1	0.125	0 --> 1
	72	1	0.091	0 --> 1
	86	1	0.087	1 --> 0
	88	1	0.074	2 --> 0
node_101 --> Onukia abliclypeus	18	1	0.200	1 ==> 2
	59	1	0.154	1 ==> 0
node_101 --> node_100	6	1	0.111	2 --> 1
	7	1	0.091	1 ==> 2
	19	1	0.105	0 ==> 1
	23	1	0.182	1 ==> 2
	53	1	0.200	0 --> 1

	56	1	0.077	0 ==> 1
	79	1	0.136	1 ==> 2
	87	1	0.095	0 --> 1
node_100 --> Paraonukia wangmoensis	17	1	0.100	1 ==> 0
	18	1	0.200	1 --> 0
	37	1	0.250	1 ==> 0
	53	1	0.200	1 --> 2
	71	1	0.094	0 ==> 1
	82	1	0.118	0 ==> 1
	83	1	0.083	1 ==> 0
	86	1	0.087	0 --> 1
	89	1	0.111	2 ==> 3
	94	1	0.167	0 ==> 1
node_100 --> Onukia flavimacula	3	1	0.130	1 ==> 2
	70	1	0.125	1 --> 0
	88	1	0.074	0 ==> 1
node_109 --> node_108	89	1	0.111	2 ==> 0
node_108 --> node_106	6	1	0.111	2 --> 1
	16	1	0.167	0 ==> 1
	48	1	0.091	0 ==> 1
	57	1	0.333	1 --> 2
	71	1	0.094	0 --> 3
	87	1	0.095	0 --> 2
node_106 --> node_105	7	1	0.091	1 ==> 0
	10	1	0.182	1 --> 0
	13	1	0.143	1 ==> 0
	14	1	0.500	1 ==> 0
	17	1	0.100	1 ==> 0
	88	1	0.074	2 --> 1
node_105 --> node_104	31	1	0.300	0 ==> 3
	48	1	0.091	1 ==> 2
	57	1	0.333	2 --> 1
	59	1	0.154	1 ==> 0
	68	1	0.200	1 ==> 0
	86	1	0.087	1 ==> 2
	89	1	0.111	0 ==> 1
node_104 --> node_103	42	1	0.250	1 ==> 2
node_103--> Evacanthus nigramericanus	49	1	0.143	0 ==> 1
	50	1	0.154	1 ==> 0
	89	1	0.111	1 ==> 3
node_103 --> Evacanthus stigmatus	1	1	0.071	1 ==> 0
	5	1	0.250	1 ==> 0
	44	1	0.133	1 ==> 2
	74	1	0.111	0 ==> 1
node_104 --> Evacanthus heimianus	10	1	0.182	0 --> 1
node_105 --> Boundarus trimaculatus	1	1	0.071	1 ==> 0
	5	1	0.250	1 ==> 0
	12	1	0.143	1 ==> 0
	19	1	0.105	0 ==> 1

	44	1	0.133	1 ==> 2
	49	1	0.143	0 ==> 1
	50	1	0.154	1 ==> 0
	71	1	0.094	3 --> 0
	83	1	0.083	1 ==> 0
	87	1	0.095	2 --> 1
	92	1	0.087	0 ==> 1
node_106 --> Evacanthus bistigmanus	46	1	0.167	0 ==> 1
	56	1	0.077	0 ==> 1
	72	1	0.091	0 ==> 1
	79	1	0.136	1 ==> 2
node_108 --> node_107	3	1	0.130	1 ==> 0
	59	1	0.154	1 ==> 0
node_107 --> Subulatus sp.n.	10	1	0.182	1 --> 2
	13	1	0.143	1 ==> 0
	18	1	0.200	1 --> 0
	69	1	0.154	0 ==> 1
	71	1	0.094	0 --> 2
node_107-->Shortcrowna flavocapitata	7	1	0.091	1 ==> 0
	17	1	0.100	1 ==> 0
	64	1	0.250	1 ==> 0
	82	1	0.118	0 ==> 1
	83	1	0.083	1 ==> 0
	84	1	0.083	0 ==> 1
	88	1	0.074	2 --> 0
node_117 --> node_116	71	1	0.094	0 --> 2
	89	1	0.111	2 --> 1
node_116 --> node_110	56	1	0.077	0 ==> 1
	88	1	0.074	0 ==> 1
node_110 --> Angustella nigricarina	48	1	0.091	0 ==> 2
	86	1	0.087	1 ==> 2
	87	1	0.095	1 --> 2
	89	1	0.111	1 --> 2
node_110 --> Diramus khaohus	74	1	0.111	0 ==> 1
	92	1	0.087	0 ==> 1
node_116 --> node_115	6	1	0.111	1 --> 0
	19	1	0.105	0 ==> 1
	71	1	0.094	2 --> 3
node_115 --> Gen.1 sp.n.	53	1	0.200	0 ==> 2
	82	1	0.118	0 ==> 2
node_115 --> node_114	72	1	0.091	0 --> 1
	79	1	0.136	1 ==> 2
	84	1	0.083	0 ==> 1
	89	1	0.111	1 --> 0
node_114 --> node_111	48	1	0.091	0 --> 1
node_111 --> Carinata ganga	1	1	0.071	1 ==> 0
	48	1	0.091	1 --> 2
	86	1	0.087	1 ==> 0
	89	1	0.111	0 --> 3

	91	1	0.125	1 ==> 2
node_111 --> Carinata flaviscutata	72	1	0.091	1 --> 0
	82	1	0.118	0 ==> 1
	83	1	0.083	1 ==> 0
	87	1	0.095	1 ==> 0
node_114 --> node_113	71	1	0.094	3 --> 0
	88	1	0.074	0 --> 1
	92	1	0.087	0 ==> 1
node_113 --> Processus bifasciatus	56	1	0.077	0 ==> 1
	57	1	0.333	1 ==> 2
	71	1	0.094	0 --> 2
	89	1	0.111	0 --> 1
node_113 --> node_112	1	1	0.071	1 ==> 0
	6	1	0.111	0 ==> 1
	91	1	0.125	1 ==> 2
node_112 --> Paraonukia ochra	46	1	0.167	0 ==> 1
	82	1	0.118	0 ==> 1
	87	1	0.095	1 ==> 2
	89	1	0.111	0 --> 3
node_112 --> Onukiades formosanus	69	1	0.154	0 ==> 1
	83	1	0.083	1 ==> 2
	84	1	0.083	1 ==> 0
	86	1	0.087	1 ==> 0
	88	1	0.074	1 --> 0
node_119 --> node_118	18	1	0.200	0 ==> 2
	59	1	0.154	1 ==> 0
node_118 --> Multiformis sp.	3	1	0.130	1 ==> 0
	10	1	0.182	1 --> 2
	12	1	0.143	1 ==> 0
	48	1	0.091	0 ==> 1
	79	1	0.136	1 --> 2
	84	1	0.083	0 ==> 1
	87	1	0.095	1 --> 2
	88	1	0.074	0 ==> 1
	89	1	0.111	2 ==> 3
node_118 --> Bundera trimaculata	17	1	0.100	1 ==> 0
	19	1	0.105	0 ==> 1
	94	1	0.167	1 ==> 0
node_120 --> Mediporus splendens	5	1	0.250	1 ==> 2
	10	1	0.182	1 --> 2
	23	1	0.182	1 ==> 2
	48	1	0.091	0 ==> 1
	53	1	0.200	0 ==> 2
	71	1	0.094	0 --> 2
	90	1	0.067	1 ==> 0
	91	1	0.125	1 ==> 0
node_120 --> Onukia muirii	6	1	0.111	2 --> 0
	79	1	0.136	1 --> 2
	82	1	0.118	0 ==> 1

	83	1	0.083	1 ==> 0
	87	1	0.095	1 --> 2
	88	1	0.074	0 ==> 1
node_122 --> Draconirvana siamensis	2	1	0.200	1 ==> 0
	7	1	0.091	1 ==> 0
	10	1	0.182	1 --> 2
	12	1	0.143	1 ==> 2
	21	1	0.333	1 ==> 2
	34	1	0.250	0 --> 1
	36	1	0.125	0 --> 1
	47	1	0.200	0 ==> 1
	69	1	0.154	0 ==> 1
	71	1	0.094	0 --> 2
	79	1	0.136	1 --> 2
	86	1	0.087	1 --> 2
node_126 --> node_125	3	1	0.130	2 --> 1
	4	1	0.167	2 ==> 0
	11	1	0.200	1 ==> 0
	19	1	0.105	0 ==> 2
	28	1	0.250	1 --> 2
	29	1	0.286	1 --> 0
	48	1	0.091	0 --> 1
	50	1	0.154	1 --> 0
	51	1	0.200	0 --> 1
	64	1	0.250	1 ==> 0
	67	1	0.273	1 ==> 0
	83	1	0.083	1 ==> 0
	96	1	0.200	1 --> 0
node_125 --> Friscanus friscanus	1	1	0.071	0 ==> 1
	59	1	0.154	1 --> 2
	79	1	0.136	1 --> 3
	86	1	0.087	1 --> 0
	89	1	0.111	0 ==> 2
	92	1	0.087	1 ==> 0
node_125 --> node_124	45	1	0.125	0 --> 1
	72	1	0.091	0 --> 1
	90	1	0.067	1 ==> 0
	91	1	0.125	2 --> 0
	95	1	0.250	1 ==> 2
node_124 --> Epiacanthus semifuscus	3	1	0.130	1 ==> 0
	6	1	0.111	0 ==> 1
	25	1	0.182	1 ==> 0
	28	1	0.250	2 --> 1
	29	1	0.286	0 --> 1
	48	1	0.091	1 --> 0
	50	1	0.154	0 --> 1
	51	1	0.200	1 --> 0
	79	1	0.136	1 --> 2
	89	1	0.111	0 ==> 3

	92	1	0.087	1 ==> 2
node_124 --> node_123	44	1	0.133	1 ==> 2
	71	1	0.094	3 --> 0
	91	1	0.125	0 --> 3
node_147 --> node_146	2	1	0.200	1 ==> 0
	23	1	0.182	2 ==> 0
	26	1	0.286	1 ==> 2
	42	1	0.250	1 ==> 0
	44	1	0.133	1 --> 0
	47	1	0.200	0 ==> 1
	72	1	0.091	0 --> 1
	79	1	0.136	1 --> 3
	87	1	0.095	0 --> 2
	88	1	0.074	0 --> 1
	91	1	0.125	2 --> 3
	98	1	0.286	0 --> 1
node_146 --> node_143	4	1	0.167	2 --> 1
	31	1	0.300	0 ==> 1
	67	1	0.273	1 ==> 2
	70	1	0.125	0 ==> 1
	90	1	0.067	1 ==> 0
node_143 --> node_127	18	1	0.200	0 ==> 1
	53	1	0.200	0 --> 1
	56	1	0.077	0 ==> 1
	65	1	0.250	0 ==> 1
	83	1	0.083	1 ==> 0
	88	1	0.074	1 ==> 2
	92	1	0.087	1 ==> 2
	93	1	0.333	0 ==> 2
node_127 --> Antillonirvana freytagi	6	1	0.111	0 ==> 1
	8	1	0.167	0 ==> 1
	12	1	0.143	1 ==> 2
	53	1	0.200	1 --> 2
	71	1	0.094	3 --> 0
node_127 --> Jassosqualus smithii	1	1	0.071	0 ==> 1
	4	1	0.167	1 --> 2
	5	1	0.250	4 ==> 0
	7	1	0.091	1 ==> 2
	19	1	0.105	0 ==> 1
	91	1	0.125	3 --> 0
node_143 --> node_142	3	1	0.130	2 --> 1
	79	1	0.136	3 --> 2
	87	1	0.095	2 --> 1
	96	1	0.200	1 --> 0
node_142 --> node_141	72	1	0.091	1 --> 0
	82	1	0.118	1 --> 0
	84	1	0.083	1 ==> 0
	88	1	0.074	1 --> 0
	91	1	0.125	3 --> 0

node_141 --> node_128	4	1	0.167	1 ==> 0
	8	1	0.167	0 ==> 1
	36	1	0.125	1 ==> 2
	89	1	0.111	0 ==> 2
	93	1	0.333	0 ==> 1
node_128 --> Yaoundea semela	3	1	0.130	1 ==> 3
	7	1	0.091	1 ==> 2
node_141 --> node_140	19	1	0.105	0 --> 1
	53	1	0.200	0 --> 1
	70	1	0.125	1 ==> 0
	86	1	0.087	0 --> 1
	92	1	0.087	1 --> 0
node_140 --> node_130	1	1	0.071	0 --> 1
	25	1	0.182	1 --> 2
	67	1	0.273	2 --> 1
	74	1	0.111	0 --> 1
	82	1	0.118	0 --> 1
	87	1	0.095	1 ==> 0
node_130 --> node_129	86	1	0.087	1 --> 2
	92	1	0.087	0 --> 1
node_129 --> Decursusnirvana excels	12	1	0.143	1 ==> 0
	25	1	0.182	2 --> 1
	34	1	0.250	1 ==> 0
	36	1	0.125	1 ==> 0
	48	1	0.091	0 ==> 1
	50	1	0.154	1 ==> 0
	53	1	0.200	1 --> 0
	56	1	0.077	0 ==> 1
	67	1	0.273	1 --> 2
	74	1	0.111	1 --> 0
node_129-->Australnirvana adelaidae	1	1	0.071	1 --> 0
	3	1	0.130	1 ==> 2
	4	1	0.167	1 ==> 2
	5	1	0.250	4 ==> 0
	7	1	0.091	1 ==> 2
	31	1	0.300	1 ==> 0
	47	1	0.200	1 ==> 0
	71	1	0.094	3 ==> 0
	79	1	0.136	2 ==> 0
	81	1	0.333	0 ==> 1
	83	1	0.083	1 ==> 0
	93	1	0.333	0 ==> 1
	99	1	0.500	0 ==> 1
node_130 --> Chudania emeiana	4	1	0.167	1 ==> 3
	19	1	0.105	1 --> 0
	37	1	0.250	0 ==> 1
	88	1	0.074	0 ==> 1
	89	1	0.111	0 ==> 3
node_140 --> node_139	44	1	0.133	0 ==> 1

	53	1	0.200	1 --> 2
	91	1	0.125	0 ==> 1
node_139 --> Convexfronta guoi	4	1	0.167	1 ==> 0
	48	1	0.091	0 ==> 1
	53	1	0.200	2 --> 3
	84	1	0.083	0 ==> 1
	86	1	0.087	1 --> 0
	87	1	0.095	1 ==> 2
	89	1	0.111	0 ==> 1
node_139 --> node_138	3	1	0.130	1 --> 3
	8	1	0.167	0 ==> 1
	36	1	0.125	1 --> 2
	55	1	0.500	2 ==> 0
	71	1	0.094	3 --> 0
	72	1	0.091	0 ==> 1
	83	1	0.083	1 ==> 0
	90	1	0.067	0 ==> 1
node_138 --> Nirvana suturalis	7	1	0.091	1 ==> 2
	12	1	0.143	1 ==> 2
	71	1	0.094	0 --> 1
	88	1	0.074	0 ==> 2
node_138 --> node_137	92	1	0.087	0 --> 1
node_137 --> node_133	70	1	0.125	0 --> 1
node_133 --> node_131	3	1	0.130	3 --> 1
	4	1	0.167	1 ==> 2
	5	1	0.250	4 ==> 0
	8	1	0.167	1 ==> 0
	36	1	0.125	2 --> 1
	86	1	0.087	1 ==> 2
node_131 --> Sophonia rosea	53	1	0.200	2 ==> 3
	87	1	0.095	1 ==> 0
	92	1	0.087	1 --> 0
node_131 --> Oniella honesta	56	1	0.077	0 ==> 1
	79	1	0.136	2 ==> 3
	83	1	0.083	0 ==> 1
	91	1	0.125	1 ==> 2
node_132 --> Concavepla sp.n.	3	1	0.130	3 --> 2
	4	1	0.167	1 ==> 0
	56	1	0.077	0 ==> 1
	87	1	0.095	1 ==> 0
	88	1	0.074	0 ==> 1
node_132 --> Sophonia sp.	6	1	0.111	0 ==> 1
	7	1	0.091	1 ==> 2
	12	1	0.143	1 ==> 2
	15	1	0.500	0 ==> 1
	19	1	0.105	1 ==> 2
	53	1	0.200	2 ==> 3
	70	1	0.125	1 --> 0
	89	1	0.111	0 ==> 1

	90	1	0.067	1 ==> 0
	92	1	0.087	1 --> 0
node_137 --> node_136	49	1	0.143	0 ==> 1
node_136 --> Ophiuchus sp.n.	3	1	0.130	3 --> 1
	6	1	0.111	0 ==> 1
	12	1	0.143	1 ==> 2
	36	1	0.125	2 --> 1
	48	1	0.091	0 ==> 1
	90	1	0.067	1 ==> 0
	91	1	0.125	1 ==> 0
	93	1	0.333	0 ==> 1
node_136 --> node_135	4	1	0.167	1 ==> 0
	7	1	0.091	1 ==> 2
	71	1	0.094	0 --> 3
	82	1	0.118	0 ==> 1
	83	1	0.083	0 ==> 1
	88	1	0.074	0 ==> 1
node_135 --> node_134	43	1	1.000	0 ==> 1
	53	1	0.200	2 ==> 3
	95	1	0.250	1 ==> 0
node_134 --> Kosasia trpica	4	1	0.167	0 ==> 2
	5	1	0.250	4 ==> 0
	8	1	0.167	1 ==> 0
	12	1	0.143	1 ==> 2
	19	1	0.105	1 ==> 2
	31	1	0.300	1 ==> 0
	48	1	0.091	0 ==> 2
	49	1	0.143	1 ==> 0
	62	1	0.250	0 ==> 1
	67	1	0.273	2 ==> 0
node_134 --> Kasunga sp.n.	3	1	0.130	3 ==> 2
node_142 --> Tortor sp.	7	1	0.091	1 ==> 0
	36	1	0.125	1 ==> 0
	44	1	0.133	0 ==> 1
	67	1	0.273	2 ==> 0
node_146 --> node_145	7	1	0.091	1 ==> 2
	45	1	0.125	0 --> 1
	92	1	0.087	1 --> 0
node_145 --> node_144	4	1	0.167	2 ==> 0
	8	1	0.167	0 ==> 1
	81	1	0.333	0 ==> 1
	87	1	0.095	2 --> 0
node_144 --> Tahura brevis	1	1	0.071	0 ==> 1
	15	1	0.500	0 ==> 1
	25	1	0.182	1 ==> 2
	31	1	0.300	0 ==> 1
	36	1	0.125	1 ==> 2
	45	1	0.125	1 --> 0
	47	1	0.200	1 ==> 0

	50	1	0.154	1 ==> 0
	53	1	0.200	0 ==> 2
	56	1	0.077	0 ==> 1
	71	1	0.094	3 --> 0
	83	1	0.083	1 ==> 0
	86	1	0.087	0 ==> 2
node_144 --> Neonirvana sp.	7	1	0.091	2 ==> 0
	19	1	0.105	0 ==> 1
	44	1	0.133	0 ==> 1
	66	1	1.000	0 ==> 1
	73	1	0.333	0 ==> 1
	82	1	0.118	1 ==> 0
	84	1	0.083	1 ==> 0
	88	1	0.074	1 --> 0
	90	1	0.067	1 ==> 0
	92	1	0.087	0 --> 1
	93	1	0.333	0 ==> 1
	95	1	0.250	1 ==> 2
	96	1	0.200	1 --> 0
node_145 --> Narecho sp.	5	1	0.250	4 ==> 1
	12	1	0.143	1 ==> 2
	19	1	0.105	0 ==> 2
	71	1	0.094	3 ==> 1
	72	1	0.091	1 --> 0
	89	1	0.111	0 ==> 2
	91	1	0.125	3 --> 1
	98	1	0.286	1 --> 0
node_150 --> node_149	5	1	0.250	4 --> 0
	7	1	0.091	1 --> 0
	12	1	0.143	1 ==> 2
	16	1	0.167	0 --> 1
	19	1	0.105	0 --> 1
	44	1	0.133	1 --> 2
	79	1	0.136	1 --> 0
	98	1	0.286	0 ==> 2
node_149 --> node_148	1	1	0.071	0 ==> 1
	2	1	0.200	1 ==> 0
	13	1	0.143	0 ==> 1
	20	1	1.000	0 ==> 1
	25	1	0.182	1 ==> 2
	26	1	0.286	1 ==> 2
	29	1	0.286	1 --> 0
	30	1	0.167	1 ==> 0
	31	1	0.300	0 ==> 2
	36	1	0.125	1 --> 2
	50	1	0.154	1 ==> 0
	59	1	0.154	2 --> 0
	60	1	1.000	0 ==> 1
	63	1	0.600	1 ==> 2

	82	1	0.118	1 ==> 0
	83	1	0.083	1 ==> 2
	90	1	0.067	1 ==> 0
	91	1	0.125	1 --> 0
	92	1	0.087	1 ==> 0
	95	1	0.250	1 ==> 0
node_148 --> Balbilus sp.	28	1	0.250	1 ==> 2
	79	1	0.136	0 ==> 3
node_148 --> Stenotortor sp.	16	1	0.167	1 --> 0
	86	1	0.087	0 ==> 2
	88	1	0.074	0 ==> 1
node_149 --> Pentoffia sp.	5	1	0.250	0 --> 3
	7	1	0.091	0 --> 2
	19	1	0.105	1 --> 2
	21	1	0.333	1 ==> 0
	41	1	0.167	0 --> 1
	45	1	0.125	0 --> 1
	48	1	0.091	1 --> 2
	64	1	0.250	1 ==> 0
	65	1	0.250	0 ==> 1
	69	1	0.154	2 --> 1
	73	1	0.333	0 ==> 1
	86	1	0.087	0 ==> 1
	87	1	0.095	0 ==> 1
	96	1	0.200	1 --> 0
	97	1	0.667	0 ==> 1
node_153 --> node_152	24	1	0.500	0 ==> 1
	32	1	0.500	0 --> 1
	33	1	0.500	1 ==> 0
	44	1	0.133	1 --> 0
	50	1	0.154	1 ==> 0
	52	1	0.400	1 ==> 0
	54	1	0.667	0 ==> 2
	55	1	0.500	2 ==> 0
	58	1	0.333	0 ==> 1
	65	1	0.250	0 ==> 1
	67	1	0.273	1 ==> 3
	74	1	0.111	0 ==> 1
	83	1	0.083	1 ==> 0
node_152 --> Alebra albostriella	21	1	0.333	1 ==> 0
	59	1	0.154	0 ==> 2
	71	1	0.094	0 --> 3
	92	1	0.087	1 ==> 0
	95	1	0.250	1 ==> 0
node_152 --> Empoasca fabae	26	1	0.286	1 ==> 0
	30	1	0.167	1 ==> 0
	51	1	0.200	0 ==> 1
	90	1	0.067	1 ==> 0
	91	1	0.125	1 --> 3

node_155 --> node_154	21	1	0.333	1 ==> 2
	38	1	1.000	0 ==> 1
	40	1	1.000	0 ==> 1
	42	1	0.250	1 ==> 2
	57	1	0.333	0 --> 1
	63	1	0.600	1 --> 3
	72	1	0.091	0 --> 1
	76	1	0.333	1 --> 0
	88	1	0.074	0 ==> 1
	92	1	0.087	1 ==> 0
	95	1	0.250	1 ==> 2
node_154 --> Phlogis sp.	1	1	0.071	0 ==> 1
	22	1	0.400	2 ==> 0
	26	1	0.286	1 ==> 2
	30	1	0.167	1 ==> 0
	36	1	0.125	0 ==> 1
	41	1	0.167	0 --> 1
	46	1	0.167	0 ==> 1
	52	1	0.400	1 ==> 2
	65	1	0.250	0 ==> 1
	89	1	0.111	0 ==> 3
node_154 --> Signoretia sp.	4	1	0.167	2 ==> 3
	5	1	0.250	4 ==> 0
	6	1	0.111	0 ==> 2
	9	1	0.333	0 ==> 1
	11	1	0.200	0 ==> 1
	23	1	0.182	1 --> 0
	29	1	0.286	0 ==> 2
	44	1	0.133	1 --> 0
	62	1	0.250	0 ==> 1
	71	1	0.094	0 --> 3
	74	1	0.111	0 ==> 1
	86	1	0.087	0 ==> 1
	90	1	0.067	1 ==> 0
	91	1	0.125	1 --> 0
	99	1	0.500	0 ==> 1
	100	1	0.333	0 ==> 1
node_158 --> node_157	1	1	0.071	0 ==> 1
	10	1	0.182	0 --> 2
	19	1	0.105	0 --> 1
	22	1	0.400	2 --> 1
	23	1	0.182	1 --> 0
	24	1	0.500	0 ==> 1
	25	1	0.182	2 --> 0
	26	1	0.286	1 ==> 0
	39	1	1.000	0 ==> 1
	52	1	0.400	1 --> 0
	88	1	0.074	0 ==> 2
	95	1	0.250	1 ==> 0

	97	1	0.667	0 --> 2
	98	1	0.286	0 --> 2
node_157 --> Equeefa castlenaui	4	1	0.167	2 ==> 3
	44	1	0.133	1 --> 2
	45	1	0.125	1 --> 0
	48	1	0.091	0 ==> 1
	49	1	0.143	0 ==> 1
	50	1	0.154	1 ==> 0
	63	1	0.600	0 ==> 2
	68	1	0.200	0 ==> 1
	85	1	0.250	1 ==> 0
	91	1	0.125	0 ==> 2
node_157 --> node_156	5	1	0.250	4 ==> 3
	34	1	0.250	0 --> 2
	52	1	0.400	0 --> 2
	56	1	0.077	0 --> 1
	71	1	0.094	0 --> 3
	79	1	0.136	0 --> 1
	83	1	0.083	1 ==> 2
	90	1	0.067	1 ==> 0
node_156 --> Coelidia sp.	6	1	0.111	0 ==> 1
	9	1	0.333	0 ==> 1
	11	1	0.200	0 ==> 1
	19	1	0.105	1 --> 0
	21	1	0.333	1 ==> 2
	31	1	0.300	0 ==> 1
	37	1	0.250	1 ==> 0
	42	1	0.250	1 ==> 2
	67	1	0.273	1 ==> 2
	89	1	0.111	0 ==> 1
	98	1	0.286	2 --> 0
node_156 --> Jikradia olitoria	3	1	0.130	0 ==> 1
	22	1	0.400	1 --> 2
	23	1	0.182	0 --> 1
	25	1	0.182	0 ==> 1
	29	1	0.286	0 ==> 2
	69	1	0.154	0 ==> 2
	74	1	0.111	0 ==> 1
	81	1	0.333	0 ==> 1
	97	1	0.667	2 --> 0
node_159 --> Neocoelidia tumidifrons	3	1	0.130	0 ==> 2
	5	1	0.250	4 ==> 0
	27	1	1.000	0 ==> 1
	34	1	0.250	0 --> 1
	36	1	0.125	0 ==> 2
	44	1	0.133	1 --> 2
	50	1	0.154	1 ==> 0
	51	1	0.200	0 --> 1
	56	1	0.077	0 --> 1

71	1	0.094	0 --> 1
77	1	1.000	0 ==> 1
78	1	1.000	0 ==> 1
79	1	0.136	0 --> 2
80	1	1.000	2 --> 3
86	1	0.087	0 --> 2
90	1	0.067	1 ==> 0

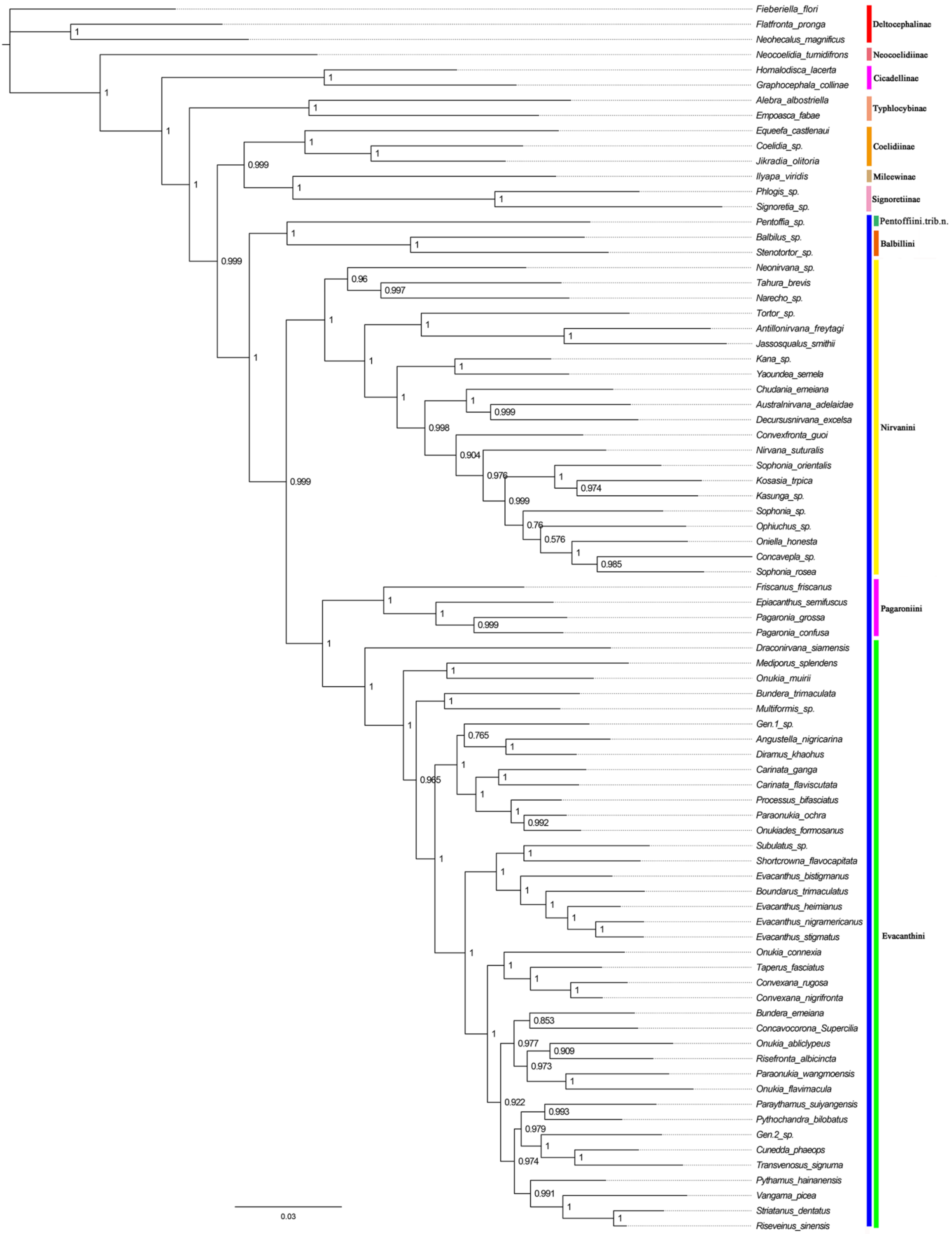

Supplementary Figure S1. Bayesian consensus tree based on combined dataset. Each node is documented which its posterior probability (PP) value.

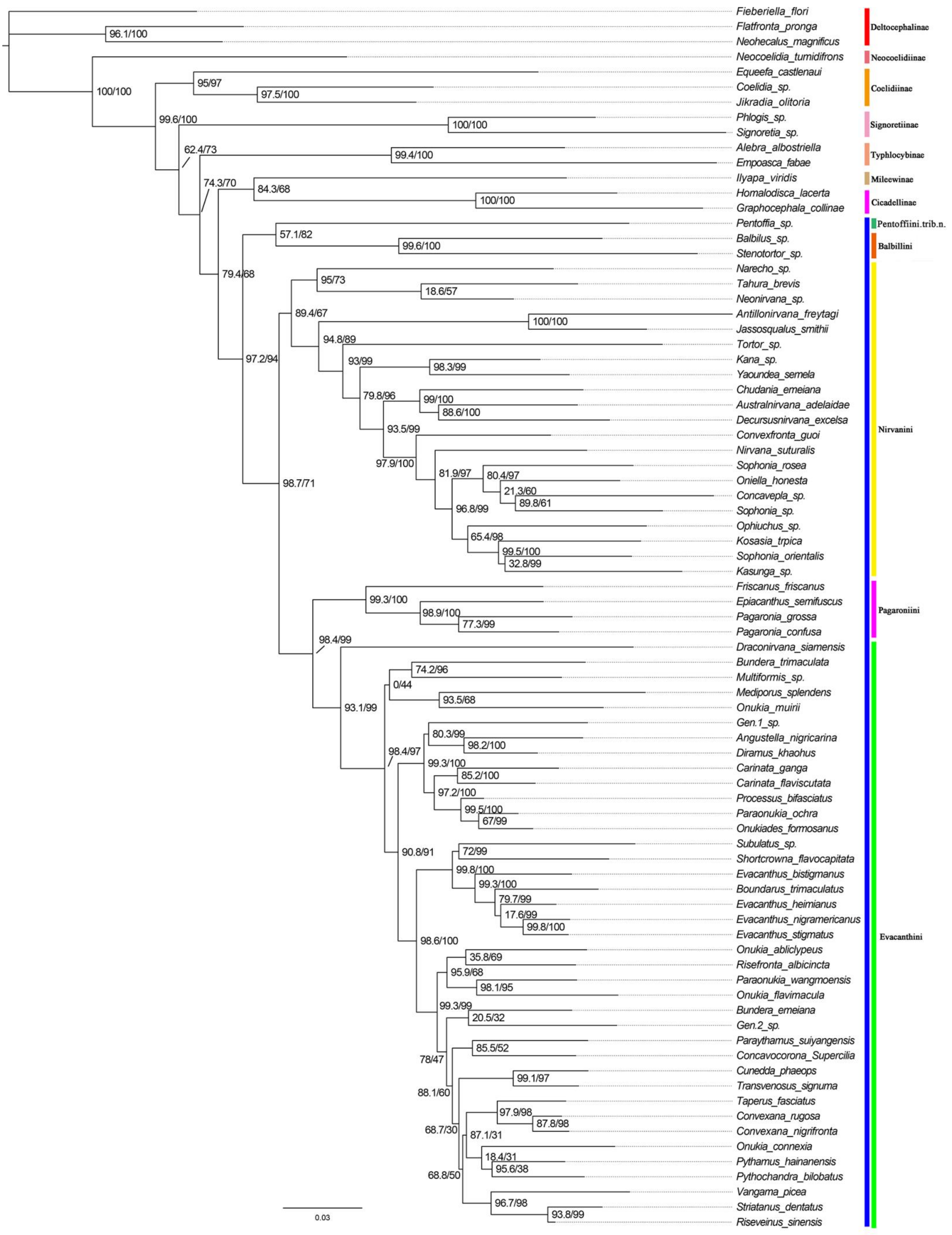

Supplementary Figure S2. Maximum-likelihood (ML) tree estimated from the molecular data only. At each node, values indicate ML support: SH-like approximate likelihood ratio test (SH-aLRT) / ultrafast bootstrap (UFB).

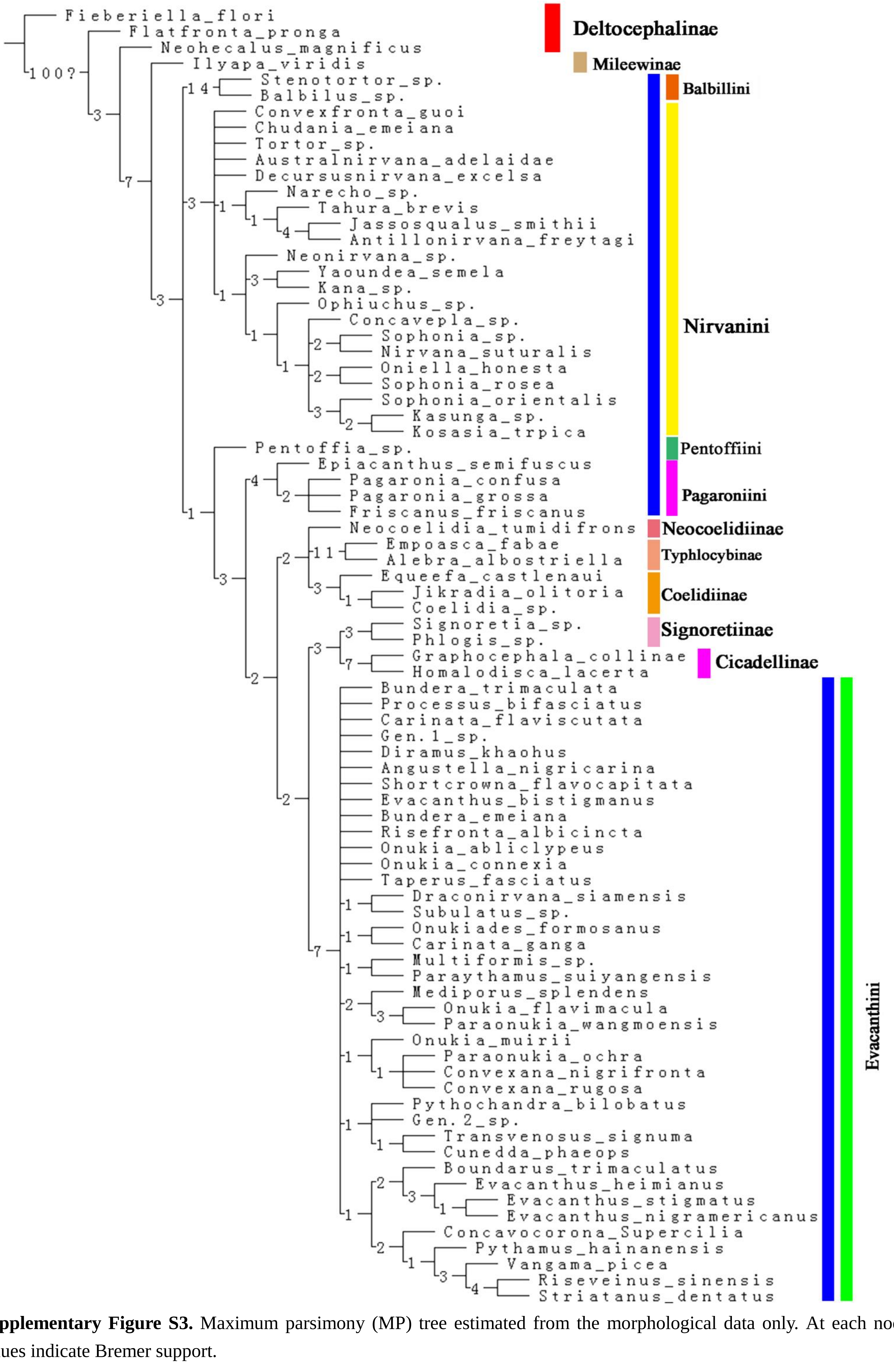

Supplementary Figure S3. Maximum parsimony (MP) tree estimated from the morphological data only. At each node, values indicate Bremer support.

Supplementary Table S1. List of taxa and DNA sequences included in the study.

Species	Family / Tribe	Voucher ID	GenBank Accession numbers				
			28S D2	28S D9-D10	H3	Wg	COI
Fieberiella flori	Deltocephalinae	LH23	AF304594*	AF304594*	KY302493	-	KR036227*
Flatfronta pronga	Deltocephalinae	WY151	KY302363	KY302428	KY302494	KY302560	KY264054
Neohecalus magnificus	Deltocephalinae	WY153	KY302364	KY302429	KY302495	KY302621	-
Homalodisca lacerta	Cicadellinae	LH97	AF304669*	AF304669*	KY302496	KY302561	KY264055
Graphocephala collinae	Cicadellinae	LH15	KY451730	KY451736	KY302497	KY302562	KY264056
Equeefa castlenaui	Coelidiinae	LH170	KY451731	KY451737	KY302498	KY302563	KY264057
Coelidia sp.	Coelidiinae	LH184	KY451732	KY451738	-	-	KY264058
Neocoelidia tumidifrons	Neocoelidiinae	LH64	AF304602*	AF304602*	KY302499	KY302623	KR040768*
Jikradia olitoria	Coelidiinae	LH24	AF304598*	AF304598*	KY302500	-	KY264059
Ilyapa viridis	Mileewinae	PR202	KY451733	KY451739	KY302501	KY302564	KY264060
Phlogis sp.	Signoretiinae	LH194	KY451734	KY451740	KY302502	KY302565	KY264061
Signoretia sp.	Signoretiinae	LH193	KY451735	KY451741	KY302503	KY302566	KY264062
Alebra albostriella	Typhlocybinae	LH98	AF304643*	AF304643*	KY302504	-	-
Empoasca fabae	Typhlocybinae	LH32	AF304640*	AF304640*	KY302505	KY302567	KY264063
Pentoffia sp.	Pentoffiini **trib.n.**	WY166	KY302365	KY302430	-	-	KY264064
Balbilus sp.	Balbillini	WY131	KY302366	KY302431	-	KY302568	KY264065
Stenotortor sp.	Balbillini	WY130	KY302367	KY302432	-	KY302569	KY264066
Tahura brevis	Nirvanini	WY128	KY302379	KY302444	-	KY302579	KY264080
Nirvana suturalis	Nirvanini	WY124	KY302380	KY302445	KY302518	KY302580	KY264081
Chudania emeiana	Nirvanini	WY127	KY302381	KY302446	KY302519	KY302581	KY264082
Concavepla sp.	Nirvanini	WY126	KY302382	KY302447	KY302520	-	KY264083
Oniella honesta	Nirvanini	WY52	KY302383	KY302448	KY302521	-	KY264084
Australnirvana adelaidae	Nirvanini	LH46	AF304635*	AF304635*	KY302522	KY302582	KY264085
Ophiuchus sp.	Nirvanini	WY144	KY302413	KY302478	KY302547	KY302609	KY264110
Sophonia sp.	Nirvanini	WY125	KY302414	KY302479	KY302548	-	KY264111
Sophonia rosea	Nirvanini	WY149	KY302415	KY302480	KY302549	KY302610	KY264112

Convexfronta guoi	Nirvanini	WY150	KY302416	KY302481	KY302550	KY302611	KY264113
Antillonirvana freytagi	Nirvanini	WY152	KY302417	KY302482	-	KY302612	KY264114
Jassosqualus smithii	Nirvanini	WY159	KY302418	KY302483	KY302551	KY302613	-
Kana sp.	Nirvanini	WY154	KY302419	KY302484	-	KY302614	KY264115
Yaoundea semela	Nirvanini	WY163	KY302420	KY302485	KY302552	KY302615	KY264116
Decursusnirvana excelsa	Nirvanini	WY157	KY302421	KY302486	KY302553	KY302616	KY264117
Sophonia orientalis	Nirvanini	WY54	KY302422	KY302487	KY302554	KY302625	KY264118
Kosasia trpica	Nirvanini	WY160	KY302423	KY302488	KY302555	KY302617	KY264119
Neonirvana sp.	Nirvanini	WY161	KY302424	KY302489	KY302556	KY302618	-
Kasunga sp.	Nirvanini	WY162	KY302425	KY302490	KY302557	-	KY264120
Tortor sp.	Nirvanini	WY165	KY302426	KY302491	KY302558	KY302619	KY264121
Narecho sp.	Nirvanini	WY164	KY302427	KY302492	KY302559	KY302620	KY264122
Pagaronia grossa	Pagaroniini	WY122	KY302378	KY302443	-	KY302577	KY264078
Pagaronia confusa	Pagaroniini	LH57	AF304605*	AF304605*	KY302517	KY302578	KY264079
Epiacanthus semifuscus	Pagaroniini	WY102	KY302412	KY302477	KY302546	KY302608	KY264109
Friscanus friscanus	Pagaroniini	LH54	AF304595*	AF304595*	KY302506	-	KY264067
Striatanus dentatus	Evacanthini	WY27	KY302368	KY302433	KY302507	KY302570	KY264068
Evacanthus nigramericanus	Evacanthini	LH82	AF304591*	AF304591*	KY302508	KY302622	KY264069
Evacanthus stigmatus	Evacanthini	WY107	KY302369	KY302434	KY302509	KY302571	KY264070
Evacanthus heimianus	Evacanthini	WY116	KY302370	KY302435	KY302510	KY302572	KY264071
Angustella nigricarina	Evacanthini	WY103	KY302371	KY302436	KY302511	-	KY264072
Carinata ganga	Evacanthini	WY104	KY302372	KY302437	KY302512	KY302624	KY264073
Diramus khaohus	Evacanthini	WY115	KY302373	KY302438	KY302513	KY302573	KY264074
Draconirvana siamensis	Evacanthini	WY113	KY302374	KY302439	-	-	KY264075
*Gen.*1 sp.	Evacanthini	WY109	KY302375	KY302440	KY302514	KY302574	KY264076
Cunedda phaeops	Evacanthini	WY105	KY302376	KY302441	KY302515	KY302575	KY264077
Transvenosus signuma	Evacanthini	WY108	KY302377	KY302442	KY302516	KY302576	-
Pagaronia grossa	Pagaroniini	WY122	KY302378	KY302443	-	KY302577	KY264078
Pagaronia confusa	Pagaroniini	LH57	AF304605*	AF304605*	KY302517	KY302578	KY264079
Onukia abliclypeus	Evacanthini	WY110	KY302384	KY302449	KY302523	-	KY264086

Taperus fasciatus	Evacanthini	WY119	KY302385	KY302450	KY302524	KY302583	KY264087
Pythamus hainanensis	Evacanthini	WY114	KY302386	KY302451	-	KY302584	KY264088
Vangama picea	Evacanthini	WY120	KY302387	KY302452	KY302525	KY302585	KY264089
Paraythamus suiyangensis	Evacanthini	WY25	KY302388	KY302453	KY302526	KY302586	KY264090
Subulatus sp.	Evacanthini	WY60	KY302389	KY302454	KY302527	KY302587	KY264091
Paraonukia wangmoensis	Evacanthini	WY63	KY302390	KY302455	KY302528	KY302588	KY264092
Risefronta albicincta	Evacanthini	WY118	KY302391	KY302456	KY302529	KY302589	KY264093
Bundera trimaculata	Evacanthini	53-1	KY302392	KY302457	KY302530	KY302590	KT183659*
Bundera emeiana	Evacanthini	23-2	KY302393	KY302458	KY302531	-	KT183628*
Concavocorona Supercilia	Evacanthini	WY46	KY302394	KY302459	KY302532	KY302591	KY264094
*Gen.*2 sp.	Evacanthini	WY132	KY302395	KY302460	-	KY302592	KY264095
Boundarus trimaculatus	Evacanthini	WY133	KY302396	KY302461	-	KY302593	KY264096
Convexana rugosa	Evacanthini	WY136	KY302397	KY302462	KY302533	KY302594	-
Mediporus splendens	Evacanthini	WY137	KY302398	KY302463	KY302534	-	KY264097
Carinata flaviscutata	Evacanthini	WY134	KY302399	KY302464	KY302535	KY302595	KY264098
Convexana nigrifronta	Evacanthini	WY135	KY302400	KY302465	KY302536	KY302596	KY264099
Riseveinus sinensis	Evacanthini	WY143	KY302401	KY302466	KY302537	KY302597	-
Processus bifasciatus	Evacanthini	WY142	KY302402	KY302467	KY302538	KY302598	-
Onukia muirii	Evacanthini	WY141	KY302403	KY302468	KY302539	KY302599	KY264100
Paraonukia ochra	Evacanthini	WY140	KY302404	KY302469	KY302540	KY302600	KY264101
Onukia flavimacula	Evacanthini	WY138	KY302405	KY302470	KY302541	KY302601	KY264102
Onukiades formosanus	Evacanthini	WY139	KY302406	KY302471	KY302542	KY302602	KY264103
Multiformis sp.	Evacanthini	WY146	KY302407	KY302472	-	KY302603	KY264104
Pythochandra bilobatus	Evacanthini	WY145	KY302408	KY302473	-	KY302604	KY264105
Shortcrowna flavocapitata	Evacanthini	WY147	KY302409	KY302474	KY302543	KY302605	KY264106
Evacanthus bistigmanus	Evacanthini	WY148	KY302410	KY302475	KY302544	KY302606	KY264107
Onukia connexia	Evacanthini	WY155	KY302411	KY302476	KY302545	KY302607	KY264108

* Numbers indicate sequences were obtained from GenBank, - indicates no sequence available.

Supplementary Table S2. PCR primers used in this study.

Primer Name	Primer sequence (5′-3′)	Primer source
LCO1490	GGTCAACAAATCATAAAGATATTGG	(Hebert et al., 2004)
HCO2198	TAAACTTCAGGGTGACCAAAAAATCA	(Hebert et al., 2004)
tRWF1_t1*	AAACTAATARCCTTCAAAG	(Park et al., 2011)
tRWF2_t1*	AAACTAATAATYTTCAAAATTA	(Park et al., 2011)
28SD2F	AGTCGKGTTGCTTGAKAGTGCAG	Dietrich et al. (2001)
28SD2R	TTCGGGTCCCAACGTGTACG	Dietrich et al. (2001)
HexAF	ATGGCTCGTACCAAGCAGACGGC	Ogden & Whiting (2003)
HexAR	ATATCCTTGGGCATGATGGTGAC	Ogden & Whiting (2003)
H3F2	AAGCAGACGGCTCGTAAATC	New design
H3R2	ATGATGGTGACYCGYTTGGC	New design
28SD9–D10F	GTAGCCAAATGCCTCGTCA	Dietrich et al. (2001)
29SD9–D10R	CACAATGATAGGAAGAGCC	Dietrich et al. (2001)
Wg1aF	GARTGYAARTGYCAYGGYATGTCTGG	Cryan et al., 2001
WgDelR1R	GTCCTGTARCCRCGKCCACAACACAT	Urban & Cryan, 2007

*: two primers combined in cocktail primer, C-tRWF.

The primer source references can be found in the text.

Supplementary Table S3. Morphological data matrix used in phylogenetic analysis

	1 – 10	11 – 20	21 – 30	31 – 40	41 – 50	51 – 60	61 – 70	71 – 80	81 – 90	91 – 100
Fieberiella flori	0 0 0 0 4 0 1 0 0 ?	1 1 0 ? 0 0 ? 0 0 0	1 2 0 0 0 1 0 1 0 0	0 1 1 2 1 0 0 0 0 0	0 1 0 2 0 1 0 1 1 1	1 1 0 0 2 1 1 1 0 0	1 0 0 0 0 0 0 0 1 0	3 0 0 1 0 0 0 0 0 1	0 0 2 0 0 0 0 1 0 1	3 0 0 1 1 0 0 0 0 0
Flatfronta pronga	0 0 0 0 4 0 1 0 0 ?	1 0 0 ? 0 0 ? 0 0 0	1 2 0 0 0 2 0 1 0 0	2 1 0 2 1 1 0 0 0 0	0 1 0 1 0 0 0 1 1 2	0 1 0 0 2 0 1 1 0 0	1 0 0 0 0 0 1 0 1 0	3 1 0 0 0 0 0 0 2 1	0 0 2 0 1 1 0 0 0 0	0 0 0 1 1 0 0 0 0 0
Neohecalus magnificus	0 0 2 0 4 0 1 0 0 ?	1 1 0 ? 0 0 ? 0 0 0	1 2 0 0 0 1 0 1 0 1	0 1 1 2 1 1 0 0 0 0	0 1 0 0 0 1 0 0 1 1	0 1 1 0 2 0 1 1 0 0	1 0 0 0 0 0 0 0 2 0	0 ? 0 0 0 0 0 0 2 1	0 0 2 0 0 2 0 0 0 1	3 1 0 1 1 0 0 2 0 1
Homalodisca lacerta	1 1 1 1 4 1 1 0 0 ?	0 ? 0 ? 0 0 ? 0 0 0	2 0 2 0 2 1 0 2 0 1	1 0 1 0 0 0 1 0 0 0	1 1 0 1 1 1 0 1 0 2	0 2 0 1 1 0 0 0 0 0	1 1 2 1 0 0 0 1 0 0	0 ? 1 0 0 0 0 0 0 0	0 0 2 0 0 0 0 0 0 1	1 1 1 1 1 0 0 0 0 1
Graphocephala collinae	0 1 1 1 4 0 0 0 0 ?	0 ? 0 ? 0 0 ? 0 0 0	2 0 2 0 1 1 0 2 0 1	0 0 1 0 0 0 1 0 0 0	1 1 0 1 1 1 0 1 0 1	0 2 0 0 0 0 0 0 0 0	1 1 1 1 0 0 1 0 1 0	0 ? 1 0 0 0 0 0 2 0	0 0 1 0 0 0 0 0 0 1	1 0 0 1 1 0 0 0 0 1
Equeefa castlenaui	1 1 0 3 4 0 0 0 0 ?	0 ? 0 ? 0 0 ? 0 1 0	1 1 0 1 0 0 0 1 0 1	0 1 1 0 1 0 1 0 1 0	1 1 0 2 0 0 0 1 1 0	0 0 0 0 2 0 0 0 0 0	1 0 2 0 0 0 1 1 0 1	0 ? 0 0 0 1 0 0 0 2	0 0 1 0 0 0 0 2 0 1	2 1 0 1 0 0 2 2 0 0
Coelidia sp.	1 1 0 2 3 1 0 0 1 2	1 1 0 1 0 0 0 0 0 0	2 1 0 1 0 0 0 1 0 1	1 1 1 2 1 0 0 0 1 0	1 2 0 1 1 0 0 0 0 1	0 2 0 0 2 1 0 0 0 0	1 0 0 0 0 0 2 0 0 1	3 0 0 0 0 1 0 0 ? 2	0 0 2 0 1 0 0 2 1 0	0 1 0 1 0 0 2 0 0 0
Neocoelidia tumidifrons	0 1 2 2 0 0 0 0 0 ?	0 ? 0 ? 0 0 ? 0 0 0	1 2 1 0 2 1 1 1 0 0	0 1 1 1 1 2 1 0 0 0	1 1 0 2 1 0 0 0 0 0	1 1 0 0 2 1 1 0 0 0	1 0 0 0 0 0 0 0 0 1	1 0 0 0 0 0 1 1 2 3	0 0 1 0 1 2 0 0 0 0	0 1 0 1 1 0 0 0 0 0
Jikradia olitoria	1 1 1 2 3 0 0 0 0 ?	0 ? 0 ? 0 0 ? 0 1 0	1 2 1 1 1 0 0 1 2 1	0 1 1 2 1 0 1 0 1 0	1 1 0 1 1 0 0 0 0 1	0 2 0 0 2 1 0 0 0 0	1 0 0 0 0 0 1 0 2 1	3 0 0 1 0 1 0 0 1 2	1 0 2 0 1 0 0 2 0 0	0 1 0 1 0 0 0 2 0 0
Ilyapa viridis	0 0 0 0 4 0 1 0 0 ?	0 ? 0 ? 0 0 ? 0 0 0	1 0 2 0 1 2 0 2 0 1	1 0 1 0 0 1 0 0 0 0	0 1 0 2 1 1 1 2 1 2	0 1 0 0 2 0 0 1 0 0	1 1 1 1 0 0 1 1 2 0	2 0 0 0 1 1 0 0 1 2	0 1 1 1 0 2 0 0 0 0	3 1 0 1 1 0 0 2 0 1
Phlogis sp.	1 1 ? 2 4 0 0 0 0 ?	0 ? 0 ? 0 0 ? 0 0 0	2 0 1 0 2 2 0 1 0 0	0 0 1 0 0 1 1 1 0 1	1 2 0 1 1 1 0 0 0 1	0 2 0 0 2 0 1 0 0 0	0 0 3 ? 1 0 1 0 0 0	0 ? 0 0 0 0 0 0 0 2	0 ? ? 0 1 0 0 1 3 1	1 0 0 1 2 0 0 0 0 0
Signoretia sp.	0 1 ? 3 0 2 0 0 1 0	1 1 0 ? 0 0 ? 0 0 0	2 2 0 0 2 1 0 1 2 1	0 0 1 0 0 0 1 1 0 1	0 2 0 0 1 0 0 0 0 1	0 1 0 0 2 0 1 0 0 0	0 1 3 ? 0 0 1 0 0 0	3 1 0 1 0 0 0 0 0 2	0 0 1 0 1 1 0 1 0 0	0 0 0 1 2 0 0 0 1 1
Alebra albostriella	0 1 0 1 4 0 0 0 0 ?	0 ? 0 ? 0 0 ? 0 0 0	0 ? ? 1 2 1 0 1 0 1	0 1 0 1 0 0 1 0 0 0	0 1 0 0 0 0 0 0 0 0	0 0 0 2 0 0 0 1 2 0	0 0 1 0 1 0 3 ? 0 0	3 0 0 1 0 1 0 0 1 2	0 1 0 0 1 0 0 0 0 1	1 0 0 1 0 0 0 0 0 0
Empoasca fabae	0 1 0 1 4 0 0 0 0 ?	0 ? 0 ? 0 0 ? 0 0 0	1 2 1 1 2 0 0 1 0 0	0 1 0 1 0 0 1 0 0 0	0 1 0 0 0 0 0 0 0 0	1 0 0 2 0 0 0 1 0 0	0 0 1 0 1 0 3 ? 0 0	0 ? 0 1 0 1 0 0 1 2	0 1 0 0 1 0 0 0 0 0	3 1 0 1 1 0 0 0 0 0
Pentoffia sp.	0 1 1 2 3 0 2 0 0 ?	1 2 0 ? 0 1 ? 0 2 0	0 ? ? 0 1 1 0 1 1 1	0 0 1 1 0 1 0 0 0 0	1 1 0 2 1 0 0 2 0 1	0 1 0 0 2 0 1 0 2 0	1 0 1 0 1 0 1 0 1 0	0 ? 1 0 0 1 0 0 0 2	0 1 1 1 0 1 1 0 0 1	1 1 0 1 1 0 1 2 0 0
Balbilus sp.	1 0 1 2 0 0 0 0 0 1	1 2 1 ? 0 1 ? 0 1 1	1 1 2 0 2 2 0 2 0 0	2 0 1 1 0 2 0 0 0 0	0 1 0 2 0 0 0 1 0 0	0 1 0 0 2 0 1 0 0 1	1 0 2 1 0 0 1 0 2 0	0 ? 0 0 0 1 0 0 3 2	0 0 2 1 0 0 0 0 0 0	0 0 0 1 0 1 0 2 0 0
Stenotortor sp.	1 0 1 2 0 0 0 0 0 1	1 2 1 ? 0 0 ? 0 1 1	1 1 2 0 2 2 0 1 0 0	2 0 1 1 0 2 0 0 0 0	0 1 0 2 0 0 0 1 0 0	0 1 0 0 2 0 1 0 0 1	1 0 2 1 0 0 1 0 2 0	0 ? 0 0 0 1 0 0 0 2	0 0 2 1 0 2 0 1 0 0	0 0 0 1 0 1 0 2 0 0
Friscanus friscanus	1 1 1 0 4 0 1 0 0 ?	0 ? 0 ? 0 0 ? 0 2 0	1 1 2 0 1 1 0 2 0 1	0 0 1 0 0 0 1 0 0 0	1 1 0 1 0 0 0 1 0 0	1 1 0 0 2 0 1 0 2 0	1 0 1 0 0 0 0 0 0 0	3 0 0 0 0 1 0 0 3 2	0 1 0 1 0 0 0 0 2 1	2 0 0 1 1 0 0 0 0 0
Striatanus dentatus	1 1 3 2 2 2 2 0 1 2	1 2 0 0 0 0 0 2 2 0	1 1 2 0 0 1 0 1 2 1	0 0 1 0 0 0 1 0 0 0	1 2 0 1 0 0 0 0 0 1	0 1 0 1 2 0 1 0 0 0	1 0 1 1 0 0 1 1 0 0	3 0 0 0 0 1 0 0 1 2	0 1 0 1 0 0 2 2 1 1	1 1 0 1 0 1 0 0 0 0
Evacanthus nigramericanus	1 1 1 2 1 1 0 0 1 0	1 1 0 0 0 1 0 1 0 0	1 1 1 0 0 1 0 1 2 1	3 0 1 0 0 0 1 0 0 0	1 2 0 1 0 0 0 2 1 0	0 1 0 0 2 0 1 0 0 0	1 0 ? 1 0 0 1 0 0 0	3 0 0 0 0 1 0 0 1 2	0 0 1 0 0 2 2 1 3 1	1 0 0 1 0 1 0 0 0 0
Evacanthus stigmatus	0 1 1 2 0 1 0 0 1 0	1 1 0 0 0 1 0 1 0 0	1 1 1 0 0 1 0 1 2 1	3 0 1 0 0 0 1 0 0 0	1 2 0 2 ? ? 0 2 0 1	0 1 0 0 2 0 1 0 0 0	1 0 ? 1 0 0 1 0 0 0	3 0 0 1 0 1 0 0 1 2	0 0 1 0 0 2 2 1 1 1	1 0 0 1 0 1 0 0 0 0
Evacanthus heimianus	1 1 1 2 1 1 0 0 1 1	1 1 0 0 0 1 0 1 0 0	1 1 1 0 0 1 0 1 2 1	3 0 1 0 0 0 1 0 0 0	1 1 0 1 0 0 0 2 0 1	0 1 0 0 2 0 1 0 0 0	1 0 ? 1 0 0 1 0 0 0	3 0 0 0 0 1 0 0 1 2	0 0 1 0 0 2 2 1 1 1	1 0 0 1 0 1 0 0 0 0
Angustella nigricarina	1 1 1 2 1 1 1 0 1 1	1 1 1 1 0 0 1 0 0 0	1 1 1 0 0 1 0 1 2 1	0 0 1 0 0 0 1 0 0 0	1 1 0 1 0 0 0 2 0 1	0 1 0 0 2 1 1 0 1 0	1 0 1 1 0 0 1 1 0 0	2 0 0 0 0 1 0 0 1 2	0 0 1 0 0 2 2 1 2 1	1 0 0 1 0 1 0 0 0 0
Carinata ganga	0 1 1 2 1 0 1 0 1 1	1 1 1 1 0 0 1 0 1 0	1 1 1 0 0 1 0 1 2 1	0 0 1 0 0 0 1 0 0 0	1 1 0 1 0 0 0 2 0 1	0 1 0 0 2 0 1 0 1 0	1 0 1 1 0 0 1 1 0 0	3 1 0 0 0 1 0 0 2 2	0 0 1 1 0 0 1 0 3 1	2 0 0 1 0 1 0 0 0 0
Diramus khaohus	1 1 1 2 1 1 1 0 1 1	1 1 1 1 0 0 1 0 0 0	1 1 1 0 0 1 0 1 2 1	0 0 1 0 0 0 1 0 0 0	1 1 0 1 0 0 0 0 0 1	0 1 0 0 2 1 1 0 1 0	1 0 1 1 0 0 1 1 0 0	2 0 0 1 0 1 0 0 1 2	0 0 1 0 0 1 1 1 1 1	1 1 0 1 0 1 0 0 0 0
Draconirvana siamensis	0 0 2 2 1 2 0 0 1 2	1 2 0 1 0 0 0 0 0 0	2 1 1 0 0 1 0 1 1 1	0 0 1 1 0 1 1 0 0 0	1 1 0 1 0 0 1 0 0 1	0 1 0 0 2 0 1 0 1 0	1 0 1 1 0 0 1 1 1 0	2 0 0 0 0 1 0 0 2 2	0 0 1 0 0 2 0 0 0 1	2 1 0 1 0 1 0 0 0 0
*Gen.*1 sp.	1 1 1 2 1 0 1 0 1 1	1 1 1 1 0 0 1 0 1 0	1 1 1 0 0 1 0 1 2 1	0 0 1 0 0 0 1 0 0 0	1 1 0 1 0 0 0 0 0 1	0 1 2 0 2 0 1 0 1 0 1	0 1 1 0 0 1 1 0 0 3	0 0 0 0 1 0 0 1 2 0	2 1 0 0 1 1 0 1 1 1	0 0 1 0 1 0 0 0 0
Cunedda phaeops	1 1 1 2 2 2 2 0 1 2	1 0 0 1 0 0 1 2 0 0	1 1 1 0 0 1 0 1 2 1	0 0 1 0 0 0 1 0 0 0	1 1 0 1 0 0 0 0 0 1	0 1 0 0 2 0 1 0 1 0 1	0 1 1 0 0 1 1 0 0 0	? 0 0 0 1 0 0 1 2 0	0 1 0 0 1 2 2 2 1 1	0 0 0 0 1 0 0 0 0

Transvenosus signuma	1112222012	1001001200	1110010121	0010001000	1201000001	0100201010 1	0110011002	0000100120	0100122211	000010000
Pagaronia grossa	011040100?	0?0?00?020	1120110201	0010001000	1102100100	1100201010 1	0100000000	?000100120	1010100003	101200000
Pagaronia confusa	011040100?	0?0?00?020	1120110201	0010001000	1102100100	1100201010 1	0100000000	?000100120	1010100003	101200000
Tahura brevis	102040210?	110?10?000	1100220111	1011020000	0000000000	0120211020 1	0110010200	?000100321	1010201013	001110100
Nirvana suturalis	003140210?	120?00?010	1100120111	1011020000	0001001001	0120001020 1	0110020201	1000100220	0000112011	001100100
Chudania emeiana	101340100?	110?00?000	1100220111	1011011000	0000001001	0110201020 1	0110010203	0010100220	1100101300	001100100
Concavepla sp.	002040110?	110?00?010	1100120111	1011020000	0001001001	0120011020 1	0110020210	?000100220	0000101011	101100100
Oniella honesta	001200100?	110?00?010	1100120111	1011010000	0001001001	0120011020 1	0110020210	?000100320	0100210012	101100100
Australnirvana adelaidae	002200200?	110?00?010	1100220111	0011010000	0000000001	0110201020 1	0110010200	?010100021	1000200000	111100110
Onukia abliclypeus	1112121012	1101001200	1110010121	0010001000	1101000001	0100201000 1	0110011010	?000100120	0100000211	000010000
Taperus fasciatus	1112102011	1111001010	1120010121	0010001000	1101000001	0100201010 1	0110011200	?000100220	0100122211	001010000
Pythamus hainanensis	1122222012	1200010020	1120010121	0010011000	1101000001	0100201010 1	0110011000	?000100120	0100121011	001010000
Vangama picea	1132222012	1200010220	1120010121	0010001000	1101000001	0101201000 1	0110011000	?011100120	1100100300	001010000
Paraythamus suiyangensis	1112222012	1111001200	1110010121	0010001000	1201000101	0100201000 1	0110011000	?000100220	0100111012	101010000
Subulatus sp.	1102121012	1101001000	1110010121	0010001000	1101000001	0100201000 1	0110011102	0000100120	0100102011	001010000
Paraonukia wangmoensis	1112112012	1101000010	1120010121	0010000000	1101000001	0120211010 1	0110011011	1000100220	1000110311	001010000
Risefronta albicincta	1112101012	1111010100	1110010121	0010001000	1101000001	0100201010 1	0110011202	0000100120	0000122211	000010000
Bundera trimaculata	1112111011	1111000210	1110010121	0010001000	1101000001	0100201000 1	0110011000	?000100120	0100110211	000010000
Bundera emeiana	1112101011	1111010100	1110010121	0010001000	1101000001	0100211010 1	0110011000	?000100120	0100022211	000010000
Concavocorona Supercilia	1102121012	1200010120	1120010121	0010001000	1101000001	0100201000 1	0110011002	0000100120	0100122211	000010000
*Gen.*2 sp.	1112222012	1011000200	1110010121	0010002000	1201000001	0100201000 1	0110011000	?000100120	0000122211	000010000
Boundarus trimaculatus	0112010010	1000010110	1110010121	0010001000	1102000110	0100202010 1	0110011000	?000100120	0000111011	101010000
Convexana rugosa	1112112011	1111000010	1110010121	0010001000	1101000001	0100201010 1	0110011000	?000100220	1010121111	001010000
Mediporus splendens	0122221012	1101001000	1120010121	0010001000	1101000101	0120201010 1	0110011002	0000100120	0100110200	001010000
Carinata flaviscutata	1112101011	1111001010	1110010121	0010001000	1101000101	0100201010 1	0110011003	0000100220	1010100011	001010000
Convexana nigrifronta	1112111011	1111001010	1110010121	0010001000	1101000101	0100201010 1	0110011000	?000100220	1010122211	001010000
Riseveinus sinensis	1132222012	1200000220	1120010121	0010001000	1201000001	0101201000 1	0110011000	?000100120	1010121311	001010000
Processus bifasciatus	1112101011	1111001010	1110010121	0010001000	1101000001	0100212010 1	0110011002	1000100220	0110111111	101010000
Onukia muirii	0122101011	1101001000	1110010121	0010001000	1101000001	0100201010 1	0110011000	?000100220	1000121211	001010000
Paraonukia ochra	0112111011	1111001010	1110010121	0010001000	1101010001	0100201010 1	0110011000	?000100220	1110121312	101010000
Onukia flavimacula	1122112012	1101001110	1120010121	0010001000	1101000001	0110211010 1	0110011000	?000100220	0100011211	000010000

Onukiades formosanus	0 1 1 2 1 1 1 0 1 1	1 1 1 1 0 0 1 0 1 0	1 1 1 0 0 1 0 1 2 1	0 0 1 0 0 0 1 0 0 0	1 1 0 1 0 0 0 0 0 1	0 1 0 0 2 0 1 0 1 0 1	0 1 1 0 0 1 1 1 0 0	? 0 0 0 1 0 0 2 2 0	0 2 0 0 0 1 0 0 1 2	1 0 1 0 1 0 0 0 0
Multiformis sp.	1 1 0 2 1 1 1 0 1 2	1 0 1 1 0 0 1 2 0 0	1 1 1 0 0 1 0 1 2 1	0 0 1 0 0 0 1 0 0 0	1 1 0 1 0 0 0 1 0 1	0 1 0 0 2 0 1 0 0 0 1	0 1 1 0 0 1 1 0 0 0	? 0 0 0 1 0 0 2 2 0	0 1 1 0 1 2 1 3 1 1	0 0 1 0 1 0 0 0 0
Pythochandra bilobatus	1 1 1 2 2 2 2 0 1 2	1 1 1 1 0 0 0 2 1 0	1 1 1 0 0 1 0 1 2 1	0 0 1 0 0 0 1 0 0 0	1 1 0 1 0 0 0 0 0 1	0 1 0 0 2 0 1 0 1 0 1	1 1 1 0 0 1 1 0 0 3	0 0 0 0 1 0 0 1 2 0	1 0 0 0 1 2 2 2 1 1	0 0 0 0 1 0 0 0 0
Shortcrowna flavocapitata	1 1 0 2 1 2 0 0 1 1	1 1 1 1 0 0 0 1 0 0	1 1 1 0 0 1 0 1 2 1	0 0 1 0 0 0 1 0 0 0	1 1 0 1 0 0 0 0 0 1	0 1 0 0 2 0 1 0 0 0 1	0 1 0 0 0 1 1 0 0 0	? 0 0 0 1 0 0 1 2 0	1 0 1 0 1 0 0 0 1 1	0 0 1 0 1 0 0 0 0
Evacanthus bistigmanus	1 1 1 2 1 1 1 0 1 1	1 1 1 1 0 1 1 1 0 0	1 1 1 0 0 1 0 1 2 1	0 0 1 0 0 0 1 0 0 0	1 1 0 1 0 1 0 1 0 1	0 1 0 0 2 1 2 0 1 0 1	0 1 1 0 0 1 1 0 0 3	1 0 0 0 1 0 0 2 2 0	0 1 0 0 1 2 2 0 1 1	0 0 1 0 1 0 0 0 0
Onukia connexia	1 1 1 2 1 0 1 0 1 1	1 1 1 1 0 0 1 0 1 0	1 1 1 0 0 1 0 1 2 0	0 0 1 0 0 0 1 0 0 0	1 1 0 1 0 0 0 0 0 1	0 1 0 0 2 0 1 0 1 0 1	0 1 1 0 0 1 1 0 0 0	? 0 0 0 1 0 0 2 2 0	0 0 0 0 1 2 2 0 1 1	1 0 1 0 1 0 0 0 0
Epiacanthus semifuscus	0 1 0 0 4 1 1 0 0 ?	0 ? 0 ? 0 0 ? 0 2 0	1 1 2 0 0 1 0 1 1 1	0 0 1 0 0 0 1 0 0 0	1 1 0 1 1 0 0 0 0 1	0 1 0 0 2 0 1 0 1 0 1	0 1 0 0 0 0 0 0 0 3	1 0 0 0 1 0 0 2 2 0	1 0 1 0 1 0 0 3 0 0	2 0 1 2 0 0 0 0 0
Ophiuchus sp.	0 0 1 1 4 1 1 1 0 ?	1 2 0 ? 0 0 ? 0 1 0	1 1 0 0 1 2 0 1 1 1	1 0 1 1 0 1 0 0 0 0	0 0 0 1 0 0 1 1 1 1	0 1 2 0 0 0 1 0 2 0 1	0 1 1 0 0 2 0 2 0 0	? 0 0 0 1 0 0 2 2 0	0 0 0 0 1 1 0 0 0 0	1 1 1 1 0 0 1 0 0
Sophonia sp.	0 0 3 1 4 1 2 1 0 ?	1 2 0 ? 1 0 ? 0 2 0	1 1 0 0 1 2 0 1 1 1	1 0 1 1 0 2 0 0 0 0	0 0 0 1 0 0 1 0 0 1	0 1 3 0 0 0 1 0 2 0 1	0 1 1 0 0 2 0 2 0 0	? 0 0 0 1 0 0 2 2 0	0 0 0 0 1 1 0 1 0 1	0 0 1 1 0 0 1 0 0
Sophonia rosea	0 0 1 2 0 0 1 0 0 ?	1 1 0 ? 0 0 ? 0 1 0	1 1 0 0 1 2 0 1 1 1	1 0 1 1 0 1 0 0 0 0	0 0 0 1 0 0 1 0 0 1	0 1 3 0 0 0 1 0 2 0 1	0 1 1 0 0 2 0 2 1 0	? 0 0 0 1 0 0 2 2 0	0 0 0 0 2 0 0 0 1 1	0 0 1 1 0 0 1 0 0
Convexfronta guoi	0 0 1 0 4 0 1 0 0 ?	1 1 0 ? 0 0 ? 0 1 0	1 1 0 0 1 2 0 1 1 1	1 0 1 1 0 1 0 0 0 0	0 0 0 1 0 0 1 1 0 1	0 1 3 0 2 0 1 0 2 0 1	0 1 1 0 0 2 0 2 0 3	0 0 0 0 1 0 0 2 2 0	0 1 1 0 0 2 0 1 0 1	0 0 1 1 0 0 1 0 0
Antillonirvana freytagi	0 0 2 1 4 1 1 1 0 ?	1 2 0 ? 0 0 ? 1 0 0	1 1 0 0 1 2 0 1 1 1	1 0 1 1 0 1 0 0 0 0	0 0 0 0 0 0 1 0 0 1	0 1 2 0 2 1 1 0 2 0 1	0 1 1 1 0 2 0 2 1 0	? 0 0 0 1 0 0 3 2 0	1 0 1 0 0 2 2 0 0 3	2 2 1 1 1 0 1 0 0
Jassosqualus smithii	1 0 2 2 0 0 2 0 0 ?	1 1 0 ? 0 0 ? 1 1 0	1 1 0 0 1 2 0 1 1 1	1 0 1 1 0 1 0 0 0 0	0 0 0 0 0 0 1 0 0 1	0 1 1 0 2 1 1 0 2 0 1	0 1 1 1 0 2 0 2 1 3	1 0 0 0 1 0 0 3 2 0	1 0 1 0 0 2 2 0 0 0	2 2 1 1 1 0 1 0 0
Kana sp.	0 0 1 0 4 0 1 1 0 ?	1 1 0 ? 0 0 ? 0 0 0	1 1 0 0 1 2 0 1 1 1	1 0 1 1 0 2 0 0 0 0	0 0 0 0 0 0 1 0 0 1	0 1 0 0 2 0 1 0 2 0 1	0 1 1 0 0 2 0 2 1 3	0 0 0 0 1 0 0 2 2 0	0 1 0 0 0 1 0 2 0 0	1 1 1 1 0 0 1 0 0
Yaoundea semela	0 0 3 0 4 0 2 1 0 ?	1 1 0 ? 0 0 ? 0 0 0	1 1 0 0 1 2 0 1 1 1	1 0 1 1 0 2 0 0 0 0	0 0 0 0 0 0 1 0 0 1	0 1 0 0 2 0 1 0 2 0 1	0 1 1 0 0 2 0 2 1 3	0 0 0 0 1 0 0 2 2 0	0 1 0 0 0 1 0 2 0 0	1 1 1 1 0 0 1 0 0
Decursusnirvana excelsa	1 0 1 1 4 0 1 0 0 ?	1 0 0 ? 0 0 ? 0 1 0	1 1 0 0 1 2 0 1 1 1	1 0 1 0 0 0 0 0 0 0	0 0 0 0 0 0 1 1 0 0	0 1 0 0 2 1 1 0 2 0 1	0 1 1 0 0 2 0 2 0 3	0 0 0 0 1 0 0 2 2 0	1 1 0 0 2 0 0 0 0 0	1 0 1 1 0 0 1 0 0
Sophonia orientalis	0 0 3 0 4 0 2 1 0 ?	1 1 0 ? 0 0 ? 0 1 0	1 1 0 0 1 2 0 1 1 1	1 0 1 1 0 2 0 0 0 0	0 0 0 1 0 0 1 0 1 1	0 1 2 0 0 0 1 0 2 0 1	0 1 1 0 0 2 0 2 0 3	1 0 0 0 1 0 0 2 2 0	1 1 0 0 1 1 1 0 1 1	1 0 1 1 0 0 1 0 0
Kosasia trpica	0 0 3 2 0 0 2 0 0 ?	1 2 0 ? 0 0 ? 0 2 0	1 1 0 0 1 2 0 1 1 1	0 0 1 1 0 2 0 0 0 0	0 0 1 1 0 0 1 2 0 1	0 1 3 0 0 0 1 0 2 0 1	1 1 1 0 0 0 0 2 0 3	1 0 0 0 1 0 0 2 2 0	1 1 0 0 1 1 1 0 1 1	1 0 1 0 0 0 1 0 0
Neonirvana sp.	0 0 2 0 4 0 0 1 0 ?	1 1 0 ? 0 0 ? 0 1 0	1 1 0 0 1 2 0 1 1 1	0 0 1 1 0 1 0 0 0 0	0 0 0 1 1 0 1 0 0 1	0 1 0 0 2 0 1 0 2 0 1	0 1 1 0 1 1 0 2 0 3	1 1 0 0 1 0 0 3 2 1	0 1 0 0 0 0 0 0 0 3	1 1 1 2 0 0 1 0 0
Kasunga sp.	0 0 2 0 4 0 2 1 0 ?	1 1 0 ? 0 0 ? 0 1 0	1 1 0 0 1 2 0 1 1 1	1 0 1 1 0 2 0 0 0 0	0 0 1 1 0 0 1 0 1 1	0 1 3 0 0 0 1 0 2 0 1	0 1 1 0 0 2 0 2 0 3	1 0 0 0 1 0 0 2 2 0	1 1 0 0 1 1 1 0 1 1	1 0 1 0 0 0 1 0 0
Tortor sp.	0 0 1 1 4 0 0 0 0 ?	1 1 0 ? 0 0 ? 0 0 0	1 1 0 0 1 2 0 1 1 1	1 0 1 1 0 0 0 0 0 0	0 0 0 1 0 0 1 0 0 1	0 1 0 0 2 0 1 0 2 0 1	0 1 1 0 0 0 0 2 1 3	1 0 0 0 1 0 0 2 2 0	1 1 1 0 0 1 1 0 0 3	1 0 1 1 0 0 1 0 0
Narecho sp.	0 0 2 2 1 0 2 0 0 ?	1 2 0 ? 0 0 ? 0 2 0	1 1 0 0 1 2 0 1 1 1	0 0 1 1 0 1 0 0 0 0	0 0 0 0 1 0 1 0 0 1	0 1 0 0 2 0 1 0 2 0 1	0 1 1 0 0 1 0 2 0 1	0 0 0 0 1 0 0 3 2 0	1 1 1 0 0 2 1 2 1 1	0 0 1 1 1 0 0 0 0
